# Age-dependent ventilator-induced lung injury: Mathematical modeling, experimental data, and statistical analysis

**DOI:** 10.1371/journal.pcbi.1011113

**Published:** 2024-02-22

**Authors:** Quintessa Hay, Christopher Grubb, Sarah Minucci, Michael S. Valentine, Jennifer Van Mullekom, Rebecca L. Heise, Angela M. Reynolds

**Affiliations:** 1 Department of Mathematics & Applied Mathematics, Virginia Commonwealth University, Richmond, Virginia, United States of America; 2 Department of Statistics, Virginia Polytechnic Institute and State University, Blacksburg, Virginia, United States of America; 3 Department of Biomedical Engineering, Virginia Commonwealth University, Richmond, Virginia, United States of America; University of Tennessee Health Science Center College of Medicine Memphis, UNITED STATES

## Abstract

A variety of pulmonary insults can prompt the need for life-saving mechanical ventilation; however, misuse, prolonged use, or an excessive inflammatory response, can result in ventilator-induced lung injury. Past research has observed an increased instance of respiratory distress in older patients and differences in the inflammatory response. To address this, we performed high pressure ventilation on young (2-3 months) and old (20-25 months) mice for 2 hours and collected data for macrophage phenotypes and lung tissue integrity. Large differences in macrophage activation at baseline and airspace enlargement after ventilation were observed in the old mice. The experimental data was used to determine plausible trajectories for a mathematical model of the inflammatory response to lung injury which includes variables for the innate inflammatory cells and mediators, epithelial cells in varying states, and repair mediators.

Classification methods were used to identify influential parameters separating the parameter sets associated with the young or old data and separating the response to ventilation, which was measured by changes in the epithelial state variables. Classification methods ranked parameters involved in repair and damage to the epithelial cells and those associated with classically activated macrophages to be influential.

Sensitivity results were used to determine candidate in-silico interventions and these interventions were most impact for transients associated with the old data, specifically those with poorer lung health prior to ventilation. Model results identified dynamics involved in M1 macrophages as a focus for further research, potentially driving the age-dependent differences in all macrophage phenotypes. The model also supported the pro-inflammatory response as a potential indicator of age-dependent differences in response to ventilation. This mathematical model can serve as a baseline model for incorporating other pulmonary injuries.

## Author summary

Pulmonary insults can prompt the need for life-saving mechanical ventilation; however, some patients can develop ventilator-induced lung injury. Prior observational data has shown increased instance of injury in older patients and differences in the inflammatory response, although further research is needed to confirm this. We performed high-pressure ventilation on young (2–3 months) and old (20–25 months) mice and observed differences in macrophage activation at baseline and lung tissue integrity following ventilation. The experimental results were used to select plausible parameter sets for a mathematical model of the inflammatory response to lung injury. Important parameters were selected based on a variety of numerical and statistical methods and results identified parameters involved in damage and repair of the epithelial cells and those controlling the pro-inflammatory response to be important in separating parameter sets based on the associated age group (young or old) and lung health after ventilation, determined by model variables. *In-silico* interventions were simulated based on these results and improvements were most influential for the parameter sets identified for the old group, specifically those with poorer lung health prior to ventilation. This mathematical model can serve as a baseline model for incorporating other pulmonary injuries.

## Introduction

A variety of inhaled pathogens and other pulmonary insults illicit an immune response that causes inflammation in the lung tissues. Intense or persistent inflammation can damage the delicate alveolar tissue and result in acute respiratory distress syndrome (ARDS). This can progress to complete respiratory failure in some cases. To increase the probability of survival, the clinical intervention for ARDS is the use of mechanical ventilation (MV) [1]. While MV is often a necessary procedure, prolonged use or misuse of the ventilator may result in ventilator-induced lung injury (VILI). The damage caused to alveolar sacs (clusters of alveolar cells) during MV can lead to volutrauma (extreme stress/strain), barotrauma (air leaks), atelectrauma (repeated opening and closing of alveoli), and biotrauma (general severe inflammatory response). The culmination of these injuries can result in ventilator dependence, multi-system organ failure, or even death [2, 3].

Although VILI can occur in patients regardless of prior lung health [2], there is a higher incidence of critical disease as well as observable differences in the inflammatory response of older individual [4–6]. Past research has shown increased risk of lung injury following ventilation for older mice [7, 8]. Most recently, infections associated with the novel coronavirus have also exhibited an increased risk of mortality and severe disease in older patients [9]. One particular study found that 6.6% of participants aged 60 years of age and older developed critical disease following a SARS-CoV-2 infection; this is approximately twelve times higher than in younger participants (0.54%) [6]. Studies have also reported increased levels of circulating inflammatory cytokines and altered macrophage function in older mice [10]. These observed discrepancies in the inflammatory response and increased rate of mortality and severe disease in elderly patients stress the need for further studies of VILI in regards to aging.

Nin *et al*. [11] found increased susceptibility and severity to pulmonary and vascular dysfunction associated with age during high tidal volume ventilation in mice. Older mice also exhibited increased levels of inflammation marked by a higher concentration of interleukin-6, a pro-inflammatory cytokine, and aspartate aminotransferase, a non-specific marker of cell injury. This intrinsic decline in the effectiveness of the innate immune response has been studied extensively [12–15]. Most notably, Dace and Apte [15] found that aging affected the polarization of macrophages, an immune cell that can exhibit a range of pro- and anti-inflammatory properties. An effective immune response relies on both a pro-inflammatory response to rid the insult of foreign cells and other debris and an anti-inflammatory response to regulate the pro-inflammatory response, promote repair, and remove debris incurred by early-response phagocytes. With aging, polarization of macrophages was observed to be skewed toward the alternatively-activated, M2, phenotype. Decreased activation of the classically-activated, M1, phenotype, generally associated with pro-inflammatory activities, could result in a decreased ability to clear infections, thus prolonging the inflammatory response and inhibiting later stages of healing.

An imbalance in the pro- and anti-inflammatory responses can cause additional complications for the individual during various injuries and insults. Macrophages in particular play a significant role in the impact of aging on the immune response [10, 16, 17]. Therefore, to develop interventions to mitigate the effects of VILI, it is important to study the immune response to lung injury and the interplay between various types of cells. We are focused on the innate immune cells, neutrophils and macrophages, their associated cytokines, and the alveolar epithelium, which consists of alveolar type I and type II cells. Alveolar type I cells make up about 95% of the alveolar surface and are primarily responsible for facilitating gas exchange. Type II cells cover the other 5% of the surface and are important in the innate immune response. In the presence of damage, these cells proliferate to repair the epithelium and can also differentiate to type I cells [18, 19]. In the present study, we examine these cells in 2–3 month old mice (young) and 20–25 month old mice (old) exposed to high-pressure MV for up to 2 hours. We present broad macrophage sub-phenotypes, M1 and M2, obtained from flow cytometry and quantitative measures of lung damage at the alveolar epithelial-endothelial barrier.

We use mathematical modeling and statistical methods to investigate the differences in the pulmonary innate immune response and predicted outcomes for the model simulations associated with either young or old experimental data. At this stage of exploration of VILI, we focus on epithelial damage and immune system interactions in young and old mice. It is difficult to clinically isolate the local epithelial and inflammatory response in the lung during VILI and often expensive to collect quality data. For this reason, we rely on *in silico* modeling of experimental data to supplement the available *in vivo* data. These *in silico* approaches provide insight into the immune response and the nonlinear dynamics of the system. The resulting analysis is used to identify important factors and generate hypotheses [20].

Minucci *et al*. [21] developed a model for VILI including major immune cell interactions involved in the inflammatory response to tissue damage and epithelial variables encompassing healthy, damaged, and dead epithelial cellular states. The current model is an expansion of the model built in Minucci *et al*. by including terms modeling epithelial barrier breakdown leading to increased cytokines and immune cells in the alveolar compartment [21]. The resulting model has 19 variables and 64 parameters. In this study we use the young and old experimental data to select plausible parameter sets and explore age-dependent outcomes and dynamics.

These mechanistic, equation-based models are often used in conjunction with statistically-based methods and models [22] to understand the possible dynamics associated with varying parameter sets. Parameter sampling and post-analysis of the mechanistic data obtained from the model are just two examples of processes that benefit from a statistical approach. To sample large parameter spaces, numerous aptly named ‘space-filling designs’ have been developed since the advent of computer experiments in the 1970s. Perhaps the most commonly used design is Latin hypercube sampling [23], but many others are used, including uniform sampling and maximin designs [24]. Many others have built off these general designs that work only for continuous data, and created variants and alternatives more specifically geared towards individual use-cases, such as the sliced Latin hypercube design [25] for categorical inputs and the fast flexible filling algorithm [26], designed for non-rectangular spaces. Machine learning algorithms have also aided in the analysis of mechanistic models. Methods such as random forest, neural networks, and principal components analysis continue to be used in congruence with mathematical models and biological systems [27–31]. These methods work well to process the large amounts of data obtained from a mechanistic model and identify abstract features of the system [22]. These algorithms can also identify nonlinear interactions between factors within the model, adding crucial insight into parameters affecting the response.

Sensitivity analysis is a useful tool for models with a large number of parameters where baseline values are unknown or difficult to measure. This approach measures changes to the model outputs from perturbations in the model inputs [32] and includes both local and global methods. In local sensitivity analysis, the change in the model output is observed when only one model parameter is varied around a selected nominal value and all other parameters are held constant. Global methods examine the sensitivity of parameters within the entire parameter space. Global techniques are usually implemented using Monte Carlo simulations, giving them the description of sampling-based methods [33]. This includes methods like Pearson correlation coefficient and partial correlation coefficients for linear trends and Spearman rank correlation coefficient and partial rank correlation coefficient for nonlinear trends with a monotonic relationship between inputs and outputs. Nonlinear non-monotonic trends require slightly different methods based on decomposition of model output variance. These methods include the Sobol method and its extended version based on (quasi) random numbers and an *ad hoc* design [34], and the Fourier amplitude sensitivity test and its extended version. The methods have been implemented in various models involving wound healing and the inflammatory response [35–39]

To determine plausible parameter sets for our model using both the young and old experimental data, we initially sampled using a beta distribution to favor lower parameter values, and then performed an iterative stochastic local search around likely candidates. The model variables were then simulated from the resulting parameter sets and model variables were compared with *in vivo* data to determine old or young presenting behavior in the resulting transients before and after ventilation. Various transient features were also calculated and analyzed including epithelial qualities and inflammatory cell quantities. Analysis of the resulting parameter sets included identifying parameters associated with young or old data, analyzing differences in lung health states between the young or old sets, and determining parameters associated with poorer lung health both including and excluding age classification. Further investigation of the identified parameter sets included local sensitivity analysis to assess model output sensitivity to variations in the parameters. The results from classification and sensitivity analysis were used to simulate pseudo-interventions to determine parameters that may be modulated to improve epithelial health during MV. This can help inform potential therapeutic targets for patients that are considered high risk before ventilation or even for patients that present signs of distress during ventilation.

## Materials and methods

### Ethics statement

Animal Research (involving vertebrate animals, embryos or tissues) Male C57BL/6 mice 8 weeks of age were purchased from Jackson Laboratory (Bar Harbor, ME). All animals were housed in accordance with guidelines from the American Association for Laboratory Animal Care and Research protocols and approved by the Institutional Animal Care Use Committee at Virginia Commonwealth University (Protocol No. AD10000465).

### Experimental materials & methods

#### Animals

Male C57BL/6 mice 8 weeks of age were purchased from Jackson Laboratory (Bar Harbor, ME). Male C57BL/6 mice 20 months of age were provided by the National Institute on Aging (Bethesda, MD). All animals were housed in accordance with guidelines from the American Association for Laboratory Animal Care and Research protocols and approved by the Institutional Animal Care Use Committee at Virginia Commonwealth University (Protocol No. AD10000465). The present study only includes male mice as male mice are the most common sex used in VILI studies [40]. Further, aged female mice were not available at the time of our study. For our future work, we will include both sexes. The results may be different in female mice as we are aware there are often inflammatory differences based on sex. For example, male mice respond with greater inflammation to lipopolysaccharides induced lung injury [41] and this may influence inflammatory results. It is unclear how this inflammation will change with age.

#### Pressure-controlled ventilator-induced lung injury model

We mechanically ventilated young (2–3 months) and old (20–25 months) C57BL/6J wild-type mice using a Scireq FlexiVent computer-driven small-animal ventilator (Montreal, Canada) and previously cited methods in Herbert *et al*. [8] with slight modifications. Mice were anesthetized, tracheotomized, and then ventilated for 5 minutes using a low pressure-controlled strategy (peak inspiratory pressure (PIP): 15 cmH_2_0, respiratory rate (RR): 125 breaths/min, positive end-expiratory pressure (PEEP): 3 cmH_2_0). Mice were then ventilated for 2 hours using a high pressure-controlled mechanical ventilation (PCMV) protocol (PIP: 35–45 cmH_2_0, RR: 90 breaths/min, and PEEP: 0 cmH_2_0). Pulmonary function and tissue mechanics were measured and collected at baseline and every hour during the 2-hour high PCMV duration using the SCIREQ FlexiVent system and FlexiWare 7 Software. A separate group of mice was anesthetized, tracheotomized, and maintained on spontaneous ventilation for 2 hours. Experimental models are limited in that they cannot be replicated for the duration of the typical time on a mechanical ventilator for humans (3 days). To adjust for this limitation, our experimental ventilator parameters are increased to cause damage that would normally be accumulated over days in a short amount of time, 2 hours.

### Tissue processing

Immediately following mechanical ventilation, the right lobes of the lung were snap frozen with liquid nitrogen, then stored at -80°C for further analysis. The left lobes of the lung were then inflated with digestion solution containing 1.5 mg/mL of Collagenase A (Roche [42]) and 0.4 mg/mL DNaseI (Roche [43]) in HBSS with 5% fetal bovine serum and 10mM HEPES and processed as previously described Yu *et al*. [44]. The resulting cells were counted, and dead cells were excluded using trypan blue. Subsets of the experimental groups were also used to collect left lobes for histological analysis.

#### Histological analysis

Lung tissue samples were embedded and stained with hematoxylin and eosin (H&E). The mean linear intercept, an index of airspace enlargement, was used to quantify relative differences in alveolar airspace area within lung histology sections. These were measured and analyzed as previously described Herbert *et al*. [8]. In our model, the high pressure mechanical ventilation does break alveolar walls and is thus quantified with airspace enlargement. If the change was due to alveolar wall creep, we would expect it to return to an undistended state upon our slow gravity fixation at 25cmH_2_O, which is not increased pressure. We did also see the presence of alveolar edema and inflammatory markers as described in [45], but the airspace enlargement gives a physical quantification of the tissue.

#### Flow cytometric analysis

Following live cell counts, 4 × 10^6^ cells per sample were incubated in blocking solution containing 5% fetal bovine serum and 2% FcBlock (BD Biosciences [46]) in PBS. The cells were then stained using a previously validated immunophenotyping panel of fluorochrome-conjugated antibodies [47] with slight modifications (See S1 Fig for a list of antibodies, clones, manufacturers, and concentrations). Following the staining procedure, cells were washed and fixed with 1% paraformaldehyde in PBS. Data were acquired and analyzed with a BD LSRFortessa-X20 [48] flow cytometer using BD FACSDiva software (BD Bioscience [49]). Histogram plots were generated using FCS Express 5 software (De Novo [50]). Compensation was performed on the BD LSRFortessa-X20 flow cytometer at the beginning of each experiment. “Fluorescence minus one” controls were used when necessary. Cell populations were identified using a sequential gating strategy that was previously developed in Misharin *et al*. [47]. The expression of activation markers was presented as median fluorescence intensity.

### Mathematical model and analysis methods

#### Model equations

**Fig 1:**
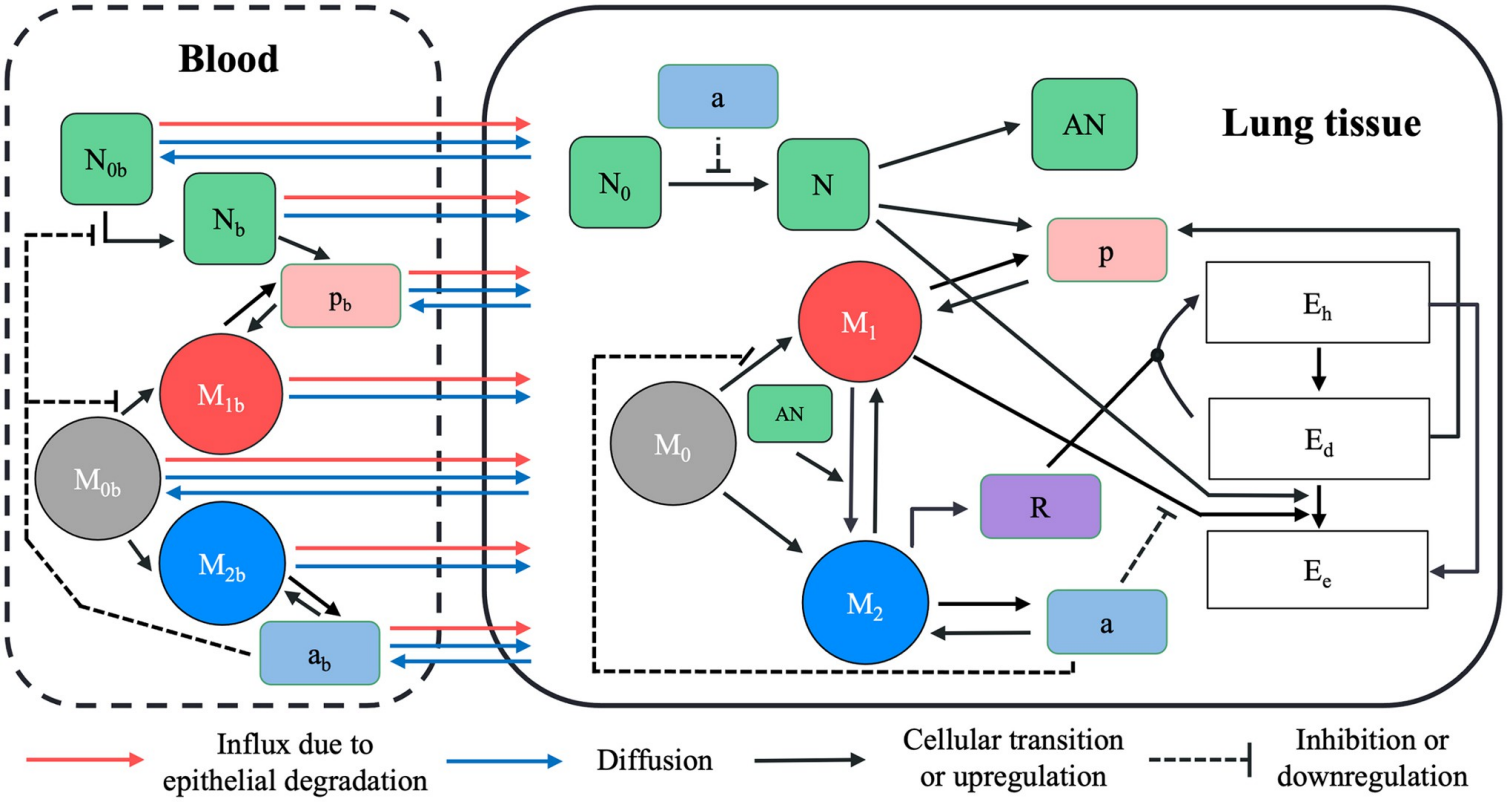
Model schematic. The model has two compartments: lung tissue and blood. The various circles and boxes represent the different inflammatory cells, mediators, and epithelial cell states. Black arrows represent upregulation or transition and black lines with bars represent inhibition or down-regulation. The blue arrows represent movement between the two compartments, either diffusion based or at a constant rate. The red arrows represent movement from the blood into the lung compartment as a result of epithelial barrier degradation.

The model uses differential equations to track the transition from a healthy lung state to a state with damage to the epithelial cells in response to ventilation. This models a direct transition from a healthy state to a damaged state at the cellular level. That is, we do not explicitly model the stress and strains at the tissue level that give rise to epithelial tissue damage. In our model damaged cells produce mediators that activate innate immune cells, neutrophils and macrophages. Immune cell influx causes additional damage to the lung epithelial cells. The epithelial cells can 1) return to a healthy state via repair, which is regulated by repair mediators, or 2) transition to the death/empty state. The portion of the population that is in the death/empty state represents the portion of the lung that needs to be replaced via proliferation of healthy epithelial cells. A full model schematic including the dynamics is given in Fig 1, model variables are in Table 1, and the parameters with brief descriptions are in Table 2.

**Table 1:** Table of model variables with descriptions.

Bloodstream	Lung	Description
	*E*_*h*_	Healthy epithelial cells
	*E*_*d*_	Damaged epithelial cells
	*E*_*e*_	Dead epithelial cells/empty space
*p*_*b*_	*p*	Pro-inflammatory mediators
*a*_*b*_	*a*	Anti-inflammatory mediators
*M*_0*b*_	*M*_0_	Naive macrophages
*M*_1*b*_	*M*_1_	M1, classically-activated macrophages
*M*_2*b*_	*M*_2_	M2 alternatively-activated macrophages
*N*_0*b*_	*N*_0_	Unactivated neutrophils
*N*_*b*_	*N*	Activated neutrophils
	*AN*	Apoptotic neutrophils
	*R*	Repair mediators

The epithelial variables *E*_*d*_, *E*_*e*_, and *E*_*h*_ are proportions, such that *E*_*h*_ + *E*_*d*_ + *E*_*e*_ = 1. The remaining variables have arbitrary units for simulation. The variables *M*_0_, *M*_1_, and *M*_2_ are used to calculate percentages for each phenotype, in order to compare to experimental flow cytometry data.

**Table 2:** Model parameters with descriptions.

Parameter	Description	Units	Young Min.	Young Max.	Old Min.	Old Max.
*ab*_∞_	Relative effectiveness of *a*_*b*_ at inhibiting *M*_0*b*_ differentiation to *M*_1*b*_	*a*-units	1.33 × 10^−4^	84.5325	3.2248	39.0498
*a*_∞_	Relative effectiveness of *a* at inhibiting *M*_0_ differentiation to *M*_1_	*a*-units	0.0108	72.5097	0.2902	57.2817
*b*_*d*_	Baseline decay of damaged cells	h^-1^	8.25 × 10^−5^	76.1677	3.0326 × 10^−3^	65.1373
*b*_*p*_	Baseline self-resolving repair of epithelial cells	h^-1^	0.8312	69.7958	0.0311	44.2681
*b*_*r*_	Baseline repair of damaged cells	h^-1^	6.96 × 10^−4^	72.1149	0.2169	47.1704
*d*_*a*_	Rate of diffusion for *a*	h^-1^	0.0125	82.1587	0.1035	67.5635
*d*_*m*0_	Rate of diffusion for *M*_0_	h^-1^	0.1717	84.7813	1.0139	59.7648
*d*_*p*_	Rate of diffusion for *p*	h^-1^	1.264 × 10^−3^	81.5292	6.8524 × 10^−3^	24.1756
*k*_*am*1_	Production rate of *a* by *M*_1*b*_ & *M*_1_	*a*-units ⋅ *M*-units^-1^ ⋅ h^-1^	0.0144	69.6048	10.9822	72.1224
*k*_*am*2_	Production rate of *a* by *M*_2*b*_ & *M*_2_	*a*-units ⋅ *M*-units^-1^ ⋅ h^-1^	6.4797 × 10^−3^	71.1447	4.6606 × 10^−3^	63.1206
*k*_*an*_	Rate at which neutrophils become apoptotic	h^-1^	0.0516	62.276	0.0403	37.3451
*k*_*anm*1_	Rate of *M*_1_ phagocytosis of *AN*	*M*-units^-1^ ⋅ h^-1^	1.73 × 10^−5^	68.8604	0.2398	54.0499
*k*_*anm*2_	Rate of *M*_2_ phagocytosis of *AN*	*M*-units^-1^ ⋅ h^-1^	1.4828 × 10^−3^	92.023	7.8663	56.181
*k*_*em*1_	Rate of phagocytosis of damaged cells by *M*_1_	*M*-units^-1^ ⋅ h^-1^	1.2397 × 10^−3^	85.7842	16.4332	98.4928
*k*_*en*_	Rate of phagocytosis of damaged cells by *N*	*N*-units^-1^ ⋅ h^-1^	2.2547 × 10^−3^	82.9453	4.1329 × 10^−3^	66.7115
*k*_*ep*_	Rate of self-resolving repair mediated by *p*	*p*-units^-1^ ⋅ h^-1^	8.4747 × 10^−3^	76.1888	0.0338	47.0804
*k*_*er*_	Rate of repair of damaged cells by *R*	h^-1^	0.0176	61.7146	5.5664 × 10^−3^	49.5429
*x*_*er*_	Regulates effectiveness of repair of damaged cells by *R* (Hill-type constant)	*R*-units	0.0217	73.0257	5.4888	67.3679
*k*_*m*0*a*_	Rate of differentiation of *M*_0_ induced by *a*	h^-1^	9.2296 × 10^−3^	75.3057	3.8383 × 10^−3^	42.3501
*x*_*m*0*a*_	Regulates effectiveness of differentiation of *M*_0_ induced by *a* (Hill-type constant)	*a*-units	1.75 × 10^−4^	83.8636	0.4768	54.2981
*k*_*m*0*ab*_	Rate of differentiation of *M*_0*b*_ induced by *a*_*b*_	h^-1^	1.757 × 10^−3^	76.8485	0.0343	68.9449
*x*_*m*0*ab*_	Regulates effectiveness of *a*_*b*_ to induce differentiation of *M*_0*b*_ (Hill-type constant)	*a*-units	5.28 × 10^−4^	73.6692	0.0149	64.5509
*k*_*m*0*p*_	Rate of differentiation of *M*_0_ induced by *p*	h^-1^	2.4 × 10^−4^	80.7515	0.6304	46.5157
*x*_*m*0*p*_	Regulates effectiveness of *p* to induce differentiation of *M*_0_ (Hill-type constant)	*p*-units	0.0996	54.3827	0.5422	33.1738
*k*_*m*0*pb*_	Rate of differentiation of *M*_0*b*_ induced by *p*_*b*_	h^-1^	2.3817 × 10^−3^	91.7174	3.3674	53.0414
*x*_*m*0*pb*_	Regulates effectiveness of *p*_*b*_ to induce differentiation of *M*_0*b*_ (Hill-type constant)	*p*-units	0.2125	70.6259	4.19 × 10^−4^	31.6476
*k*_*man*_	Rate of *M*_1_ switch to *M*_2_ by *AN*	*M*-units ⋅ *N*-units^-1^	1.0217 × 10^−3^	85.4308	0.9625	58.4498
*k*_*mne*_	Rate of collateral damage to epithelial cells by macrophages and neutrophils	h^-1^	0.0144	87.4140	4.3902	55.8505
*x*_*mne*_	Regulates effectiveness of macrophages and neutrophils to damage epithelial cells (Hill-type constant)	(*M* + *N*)-units	0.0895	68.5317	3.05 × 10^−3^	10.2302
*k*_*n*_	Rate of infiltration of *N*_*b*_ to lung	h^-1^	0.0342	92.8295	0.8702	70.1239
*k*_*n*0*pb*_	Rate of activation of *N*_*b*_ induced by *p*_*b*_	h^-1^	5.4571 × 10^−3^	79.0613	0.0686	71.3452
*x*_*n*0*pb*_	Regulates effectiveness of *p*_*b*_ to induce activation of *N*_*b*_	*p*-units	4.83 × 10^−3^	80.1988	0.0708	28.0339
*k*_*pe*_	Production rate of *p* by *E*_*d*_	*p*-units ⋅ h^-1^	5.0833	78.5846	0.0576	45.4633
*k*_*pm*1_	Production rate of *p* by *M*_1_ & *M*_1*b*_	*p*-units ⋅ *M*-units^-1^ ⋅ h^-1^	1.6489 × 10^−3^	75.4175	0.0208	68.4131
*k*_*pn*_	Production rate of *p* and *p*_*b*_ by neutrophils	*p*-units ⋅ *N*-units^-1^ ⋅ h^-1^	3.62 × 10^−5^	75.2844	0.0167	37.4531
*k*_*rm*2_	Production rate of *R* by *M*_2_	*R*-units ⋅ *M*-units^-1^ ⋅ h^-1^	2.0303 × 10^−3^	76.6348	7.235 × 10^−3^	42.9838
*μ*_*a*_	Decay rate of *a*	h^-1^	8.7545 × 10^−3^	89.7891	0.0964	58.2379
*μ*_*ab*_	Decay rate of *a*_*b*_	h^-1^	2.45 × 10^−5^	70.0997	2.2509	44.3072
*μ*_*m*0_	Decay rate of *M*_0_	h^-1^	1.2771 × 10^−3^	86.2183	18.1666	57.0514
*μ*_*m*0*b*_	Decay rate of *M*_0*b*_	h^-1^	5.0617 × 10^−3^	66.3658	0.0550	46.2847
*μ*_*m*1_	Decay rate of *M*_1_	h^-1^	0.1494	83.7439	0.0200	37.3965
*μ*_*m*1*b*_	Decay rate of *M*_1*b*_	h^-1^	6.7474 × 10^−3^	86.8009	1.8930	54.6995
*μ*_*m*2_	Decay rate of *M*_2_	h^-1^	0.3748	78.7343	1.0916	32.3682
*μ*_*m*2*b*_	Decay rate of *M*_2*b*_	h^-1^	1.1642 × 10^−3^	83.7154	0.0848	90.4447
*μ*_*n*_	Decay rate of *N*	h^-1^	3.57 × 10^−4^	74.7403	0.0170	19.7722
*μ*_*nb*_	Decay rate of *N*_*b*_	h^-1^	4.9563 × 10^−3^	82.5759	2.1406 × 10^−3^	51.4149
*μ*_*n*0*b*_	Decay rate of *N*_0*b*_	h^-1^	0.0109	73.707	8.1144 × 10^−3^	49.572
*μ*_*p*_	Decay rate of *p*	h^-1^	1.97 × 10^−4^	71.0144	0.1147	42.6670
*μ*_*pb*_	Decay rate of *p*_*b*_	h^-1^	3.7486 × 10^−3^	66.7341	7.5471 × 10^−3^	16.8999
*μ*_*R*_	Decay rate of *R*	h^-1^	1.4206	77.6702	3.6178	23.4071
*s*_*a*_	Source rate of background *a*_*b*_	*a*-units ⋅ h^-1^	0.0315	90.5490	8.8694	81.6087
*s*_*d*_	Rate of damage from ventilator	h^-1^	1.6823	82.2663	0.9770	66.8206
*s*_*m*_	Source rate of *M*_0*b*_	*M*-units ⋅ h^-1^	0.0649	83.6374	5.4517	61.3312
*s*_*n*_	Source rate of *N*_0*b*_	*N*-units ⋅ h^-1^	0.0651	86.7327	4.683	66.2144
*s*_*p*_	Source rate of background *p*_*b*_	*p*-units ⋅ h^-1^	0.1903	60.7942	4.9828	92.3764
*k*_ee_*	Rate of inflammatory cell and mediator leakage into alveolar compartment	h^-1^	2.0545 × 10^−3^	79.59	9.0485	42.4358
*x*_ee_*	Regulates effectiveness of inflammatory cell leakage into alveolar compartment (Hill-type constant)	No Units- Normalized Variable	0.75	0.75	0.75	0.75
*x*_eem_*	Regulates effectiveness of inflammatory mediator leakage into alveolar compartment (Hill-type constant)	No Units- Normalized Variable	0.5	0.5	0.5	0.5
*k*_*m*1_*	Rate of *M*_1_ infiltration into alveolar compartment	h^-1^	1.8228	74.9223	25.1536	60.8061
*k*_*m*2_*	Rate of *M*_2_ infiltration into alveolar compartment	h^-1^	4.6 × 10^−4^	91.5074	7.1409	54.7490
*k*_*n*0p_*	Rate of activation of *N* induced by *p* in the alveolar compartment	h^-1^	0.1285	85.1315	0.0144	46.1535
*x*_*n*0p_*	Regulates effectiveness of *p* to induce *N* activation in the alveolar compartment	*p*-units	0.0355	65.0746	2.0399	45.9973
*μ*_an_*	Decay rate of *AN*	h^-1^	1.2156 × 10^−3^	81.2026	0.0192	79.2533
*μ*_*n*0_*	Decay rate of *N*_0_	h^-1^	0.0393	85.9090	0.0119	48.7474

Parameters indicated with an asterisk (*) are novel to this iteration of the model and were not utilized in the Minucci *et al*. [21] model. Model variable units are arbitrary and indicated as related to the general cell type. Therefore, *N*-units represent neutrophils, *M*-units represent all phenotypes of macrophages, *p*-units represent pro-inflammatory mediators, *a*-units represent anti-inflammatory mediators, and *R*-units represent repair mediators. The time unit is hours, *h*. Parameter ranges are determined by the maximum and minimum value achieved by each parameter over the plausible parameter sets associated with young and old experimental data. The process for obtaining these ranges is explained in the following sections.

We account for macrophage phenotype on a population level. Therefore, our variables track the overall level of M1 type activity (classically activated) versus M2 type activity (alternatively activated). Activation of naive macrophages M0 by pro-inflammatory mediators (PIMs) give rise to the M1 phenotype, phagocyte cells producing PIMs and anti-inflammatory mediators (AIMs). M1 cells phagocytize both damaged epithelial cells and apoptotic neutrophils. Neutrophils also phagocytize damaged epithelial cells and produce PIMs.

Conversely, activation of naive cells by AIMs gives rise to the M2 phenotype, producing AIMs and repair mediators. M1 cells can transition to M2 cells in response to phagocytising apoptotic neutrophils. The full equations for our model are in Appendix S1–S6 Eqs. The main reference for the equation derivations are given in Minucci *et al*. [21], since our current model is an adaptation of that model.

The main change from Minucci *et al*. [21] in this model was the introduction of a breakdown in the barrier integrity that leads to an increase in inflammatory cell and mediator movement between the systemic blood compartment into the lung space that is not diffusion driven. We illustrate this with an immune cell equation, the lung *M*_0_ equation (Eq 1), and a mediator equation, the lung PIMs (*p*, Eq 2), but this type of term occurs for all the immune cells and mediators (see Appendix S1–S5 Eqs). The second to last term in the *M*_0_ equation (Eq 1) is a nonlinear Hill- type term that allows naive cells to move from the blood into the lung when the epithelial lung barrier is degraded significantly. Therefore, the term is dependent on *E*_*e*_ with the parameter *x*_*ee*_ controlling the level of *E*_*e*_ at which this term achieves its half max. *x*_*ee*_ is fixed to 0.75 for all immune cell equations. The second to last term of the *p* equation (Eq 2) has the same form but with the parameter defined as *x*_*eem*_ which is fixed to 0.5 for all mediator equations. These parameters are fixed at these levels to ensure that the smaller mediators pass through the degraded alveolar-capillary barrier at lower value of *E*_*e*_ than the immune cells. The values of *x*_*ee*_ and *x*_*eem*_ at this point create a partitioning of the range of values for *E*_*e*_, [0, 1] which is a first step to mapping the epithelial variables to clinically relevant lung injuries, such as pulmonary edema. These values were chosen so that *x*_*eem*_ is smaller than *x*_*ee*_ and that substantial damage (half of the range) is needed before reaching the half max for the mediator influx term. Mediator flux is associated with severe lung damage and immune cell leakage into the lung due to barrier disruption which would lead to ventilation failure as the alveoli fill with fluid instead of air. The third from last term in both of these equations model the diffusion driven infiltration between compartments.

The first term in the *M*_0_ equation (Eq 1) models the activation of the naive macrophages into the M1 and M2 phenotypes with down-regulation of the M1 phenotype via inhibition by AIMs represented by the variable *a*. The first three terms of the *p* equation (Eq 2) models production of PIMs by damaged epithelial cells, M1 cells (inhibited by *a*) and neutrophils, respectively. The last term in both equation models intrinsic decay.

$$\begin{array}{cc} \frac{dM_{0}}{dt}= & -M_{0}[\underset{\text{Differentiation}\;\text{to}\;\text{M1}\;\text{via}\;\text{PIMs}}{\underset{︸}{\left(\frac{k_{m0pb}p_{b}^{2}}{x_{m0pb}^{2}+p_{b}^{2}}\right)}}\underset{\text{Inhibition}\;\text{by}\;\text{AIMs}}{\underset{︸}{\left(\frac{1}{1+{\left(\frac{a}{a_{\infty }}\right)}^{2}}\right)}}+\underset{\text{Differentiation}\;\text{to}\;\text{M2}}{\underset{︸}{\left(\frac{k_{m0a}a^{2}}{x_{m0a}^{2}+a^{2}}\right)}}]\\ & -\underset{\text{Diffusion}}{\underset{︸}{d_{m0}\left(M_{0}-M_{0b}\right)}}+\underset{\text{Leak}\;\text{into}\;\text{lung}}{\underset{︸}{M_{0b}\frac{k_{ee}E_{e}^{4}}{x_{ee}^{4}+E_{e}^{4}}}}-\underset{\text{Decay}}{\underset{︸}{\mu_{M_{0}}M_{0}}} \end{array}$$ (1)

$$\begin{array}{cc} \frac{dp}{dt}= & \underset{\text{Production}\;\text{via}\;\text{ep.}\;\text{damage}}{\underset{︸}{k_{pe}E_{d}}}+\underset{\text{Production}\;\text{via}\;\text{M1}}{\underset{︸}{k_{pm1}M_{1}}}\underset{\text{Inhibition}\;\text{by}\;\text{AIMs}}{\underset{︸}{\left(\frac{1}{1+{\left(\frac{a}{a_{\infty }}\right)}^{2}}\right)}}+\underset{\text{Production}\;\text{via}\;\text{neutrophils}}{\underset{︸}{k_{pn}N}}\\ & -\underset{\text{Diffusion}}{\underset{︸}{d_{p}\left(p-p_{b}\right)}}+\underset{\text{Leak}\;\text{into}\;\text{lung}}{\underset{︸}{p_{b}\frac{k_{ee}E_{e}^{4}}{x_{eem}^{4}+E_{e}^{4}}}}-\underset{\text{Decay}}{\underset{︸}{\mu_{p}p}} \end{array}$$ (2)

#### Sampling and parameter selection

The model variables were simulated using MATLAB (R2021a) [51] to determine plausible *in silico* parameter sets associated with each experimental group. Plausible parameter sets were those where, 1) non-ventilated simulated variables reached a numerical steady state, 2) the associated steady state value fell within the range of *in vivo* data for either the young or old data at *t* = 0 hours, and 3) ventilated simulations starting from steady state fell within the range of the associated young or old data at *t* = 2 hours. During this selection process *M*_0_, *M*_1_, *M*_2_, and *E*_*e*_ were compared to the experimental data for unactivated macrophages, classically-activated macrophages, alternatively-activated macrophages, and airspace enlargement, respectively.

**Fig 2:**
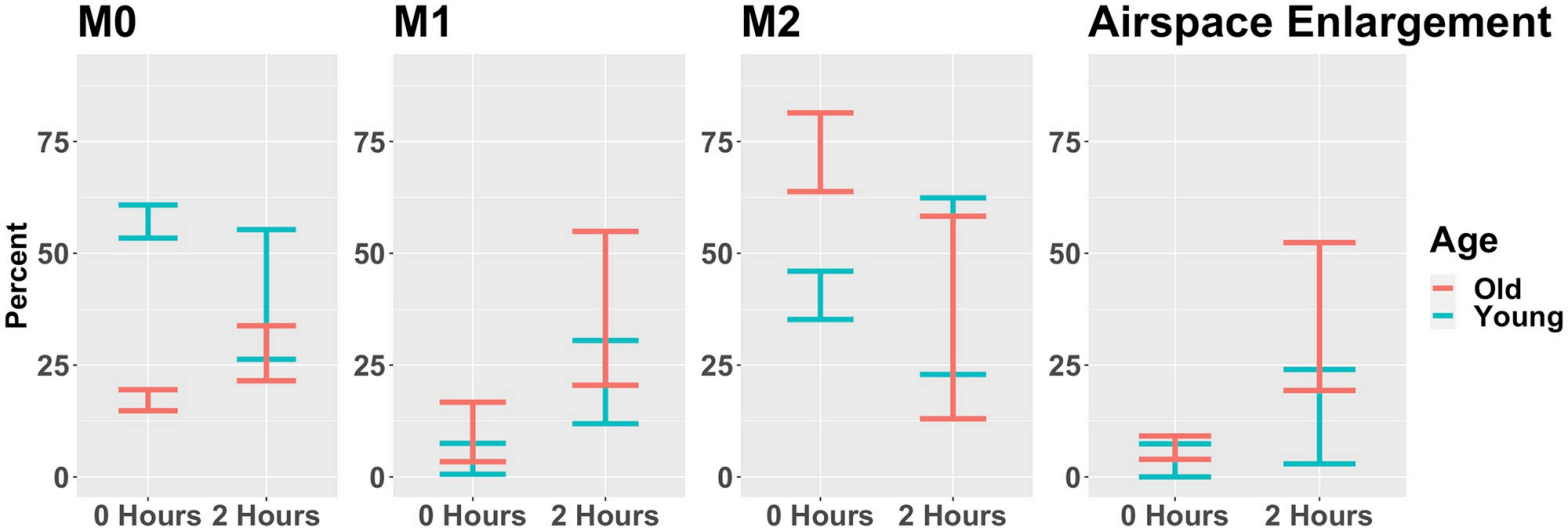
Summary of data ranges used to select plausible parameter sets. The plot gives the range of experimental values for M0, M1, and M2 macrophages and airspace enlargement used to assess the numerical simulations for biological plausibility. Each range consists of 3–6 experimental observations since some data points were excluded as outliers. Outliers were defined as those being more than two standard deviations outside the mean.

The data ranges used for each experimental group and cell are plotted in Fig 2. Note from Fig 2 some data ranges overlap significantly, but given that there is separation in the M0 and M2 data for time zero, there are no parameter sets that satisfy both young and old conditions.

Model variables were simulated for each parameter set to reach numerical steady-state to ensure that the dynamics observed during ventilation were only a product of the ventilation and not a result of the variables attempting to reach steady state. For these simulations, the parameter *s*_*d*_, a parameter used to describe the damage caused by ventilation, was set to zero. Each parameter set was simulated starting at an initial condition where all model variables were set to zero excluding M1 macrophages in the lung and healthy epithelial cells which were set to 50 and 1, respectively, initiating a return to steady state from an inflammatory insult. Simulations were run for 800 hours and classified as achieving numerical steady state if the Euclidean norm of the difference between the end values of the variables and the value at each numerical time step within the last 20 hours of simulation was less than 0.001. If the model variables did not reach steady state, the model was simulated starting at an additional initial condition where all model variables were set to zero excluding healthy epithelial cells and damaged epithelial cells which were set to 0.75 and 0.25, respectively, initiating a return to steady state from an epithelial damage-induced insult. Parameter sets that did not produce model transients that reach steady state for either initial condition were excluded from further sampling and analysis. Variable transients that achieved steady state and fell within the range of either the young or old initial data values were then simulated starting at their numerical steady state for a total of 200 hours with ventilation occurring during the first two hours (*s*_*d*_ > 0) to observe transient behavior during and after ventilation. The resulting model variables were then compared to the data at 2 hours and the respective parameter sets were classified as young or old depending on their ability to satisfy the respective data ranges. Those that did not fall within the range of either the young or the old data at both time points were excluded from the final accepted parameter sets.

Parameter sets for this process were found using a three-step parameter sampling process in R [52]. In the initial step, a large number of samples were generated using a scaled *Beta*(1, 3) distribution with a large scale parameter to sample between 0 and 120 (to include multiple orders of magnitude). Parameter sets with associated transients that fell within the range of the data for any of the macrophage variables or *E*_*e*_ at either time point for either age group were used to define the ranges of each parameter during the next stage of sampling. Uniform sampling was used over this refined space for the second stage. The final step was iterative stochastic local search; using four iterations, successively restricting the space by adding more components of the criteria until in the end all remaining sets matched every criterion for one of the age groups.

#### Classification, regression and sensitivity

Several methods were fitted on various subsets of the parameter sets which fell in the range for either the young or old data. A suite of model fitting algorithms were applied using the R package H2O [53], including Generalized Linear Models, Distributed Random Forests, Gradient Boosting Machines, and Neural Networks. The best model in terms of 5-fold cross-validated F1 score (for classification) or root mean squared error (for regression) was chosen and the variable importance values were calculated for each. In classification methods, the importance values rank the relative importance of each data feature in the defined statistical model. Thus, an importance value is calculated for each parameter based on their relative influence in separating the parameter sets into the young or old associated groups. In order to justify the importance metric for classification models where the data was not balanced (e.g., predicting young versus old), the same models were fitted on down-sampled data with balanced labels.

Local sensitivity analysis was also used to measure the relative sensitivity of the model parameters for each experimental group. The methods were implemented using the SimBiology package available in MATLAB [51] which calculates the time-dependent derivatives of the model sensitivity to each parameter evaluated at specified time points. Details about the calculations performed can be found in Martins et al. [54]. Default settings were used for the sensitivity matrix. The overall relative sensitivities for each parameter were calculated by taking the root mean square of the sensitivity values at the chosen time points for the chosen variables. The resulting values for each parameter were then normalized by scaling each to the maximum overall sensitivity value in each group.

## Results

### Experimental results

A large difference was observed in M0 cells at baseline between young and old mice (Fig 2). There were increases in M1 marker expression in the alveolar macrophage populations (Fig 2) from both the young and old mice after 2 hours of PCMV. M2 cells were increased at baseline in the old mice, with overlapping ranges in the PCMV mice. High PCMV enlarged the airspace in both young and old mice. The mean linear intercept, which is an index of airspace enlargement, was quantified to further assess the extent of injury (Fig 2). There was significantly increased airspace enlargement in the old PCMV group compared to the young and old non-ventilated controls. These findings suggest that there was a substantial generation of acute lung injury in both the young and old age groups; however, the severity appears to intensify with the old mice.

### *In silico* results

#### Plausible parameter sets and transients

**Fig 3:**
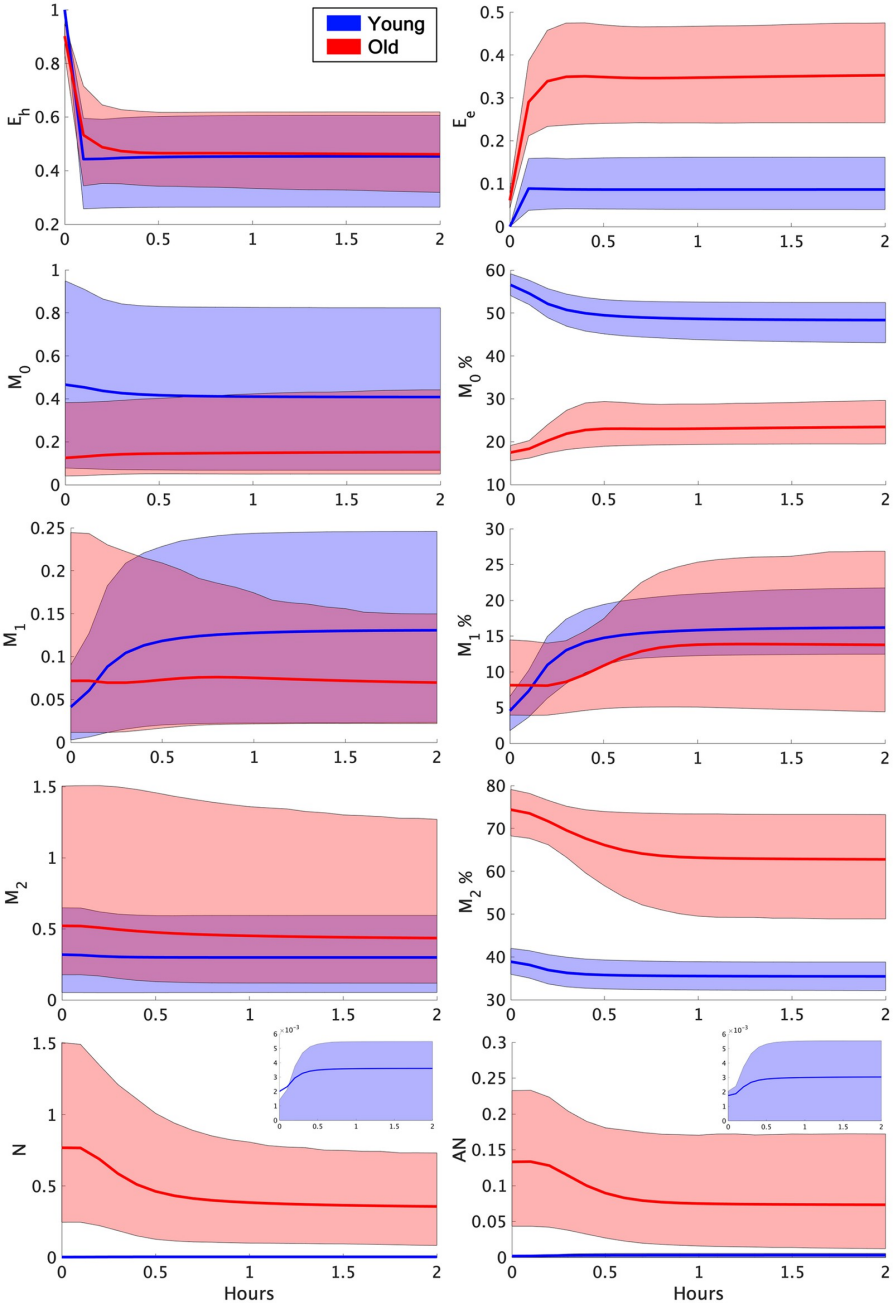
Average young and old transients. The solid blue and red lines plotted within the corresponding shaded bands represent the mean response for the transients associated with each experimental group at each time point. The borders of the surrounding bands encompass the 10th and 90th percentile at each time point. M_0_%, M_1_%, and M_2_% represent the percentage of the macrophage activity that is M0, M1, and M2, respectively. These percentages along with the *E*_*e*_ variable were validated by the experimental data at 0 hours and 2 hours. Due to differences in scale, the variables *N* and *AN* also include overlays with just the young mean and percentiles plotted.

Iterative sampling used for the large sampling space produced 19,202 plausible parameter sets associated with either the young or old experimental data. Of these sets, 17,477 were associated with the young data and 1,725 were associated with the old data. Average model behavior for the variables *E*_*h*_, *E*_*e*_, *M*_0_, *M*_1_, *M*_2_, *N*, and *AN* are shown in Fig 3. Additionally, the percentage of the macrophage activity that was *M*_0_, *M*_1_, or *M*_2_ were plotted.

**Fig 4:**
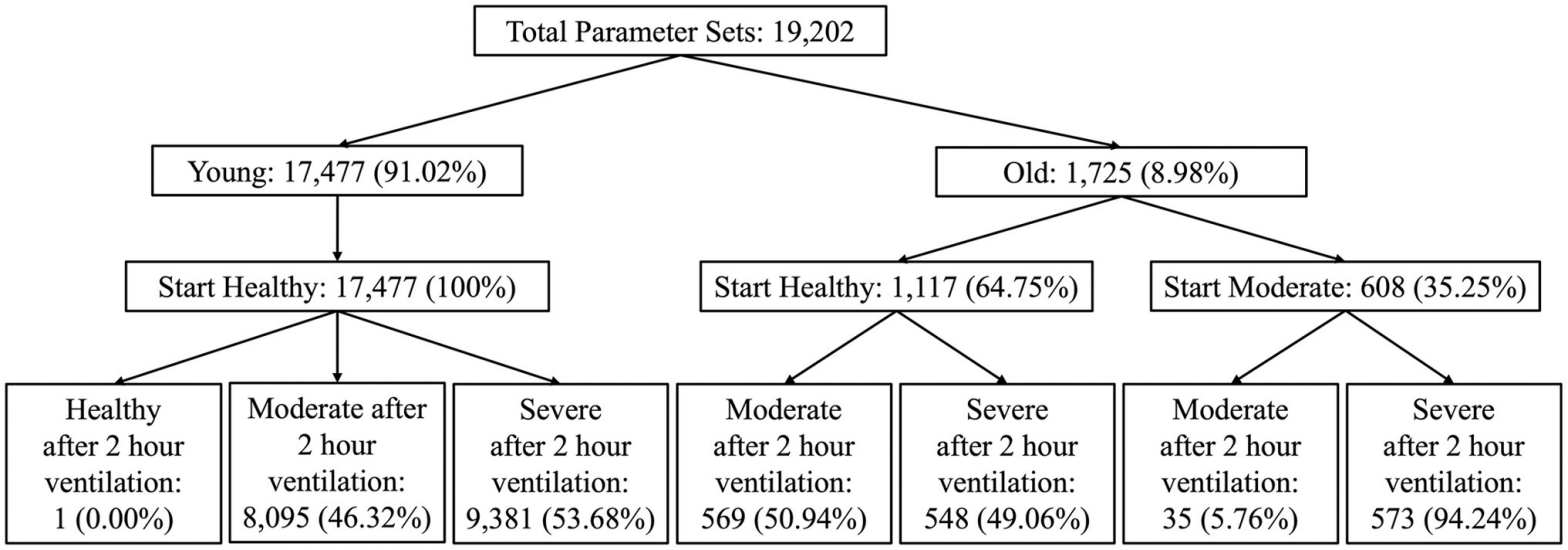
Plausible parameter sets separated by age group and epithelial health. Separation of plausible sets was made using the *E*_*h*_ variable proportion before and after 2 hours of ventilation. Transients were classified as healthy when *E*_*h*_ > 0.9, moderate when 0.5 < *E*_*h*_ < 0.9, or severe when *E*_*h*_ < 0.5. Corresponding percentages were calculated at each level of separation in the flowchart as a percentage of the previous bin.

Parameter sets were separated using the proportion of their associated *E*_*h*_ variable value before ventilation (*t* = 0) and directly after the 2 hour ventilation (*t* = 2). Parameter sets were defined at each selected time point as healthy when *E*_*h*_ > 0.9, moderate when 0.5 < *E*_*h*_ < 0.9, and severe when *E*_*h*_ < 0.5. The resulting groups are shown in Fig 4.

This separation scheme created a natural division in the plausible parameter sets into the following groups by age and ventilation response: young sets that started healthy and became moderate after ventilation (Young H2M), young sets that started healthy and became severe after ventilation (Young H2S), old sets that started healthy and became moderate after ventilation (Old H2M), old sets that started healthy and became severe after ventilation (Old H2S), old sets that started moderate and stayed moderate after ventilation (Old M2M), and old sets that started moderate and became severe after ventilation (Old M2S). We will exclude the one young set that remained healthy after ventilation due to the sample size.

**Fig 5:**
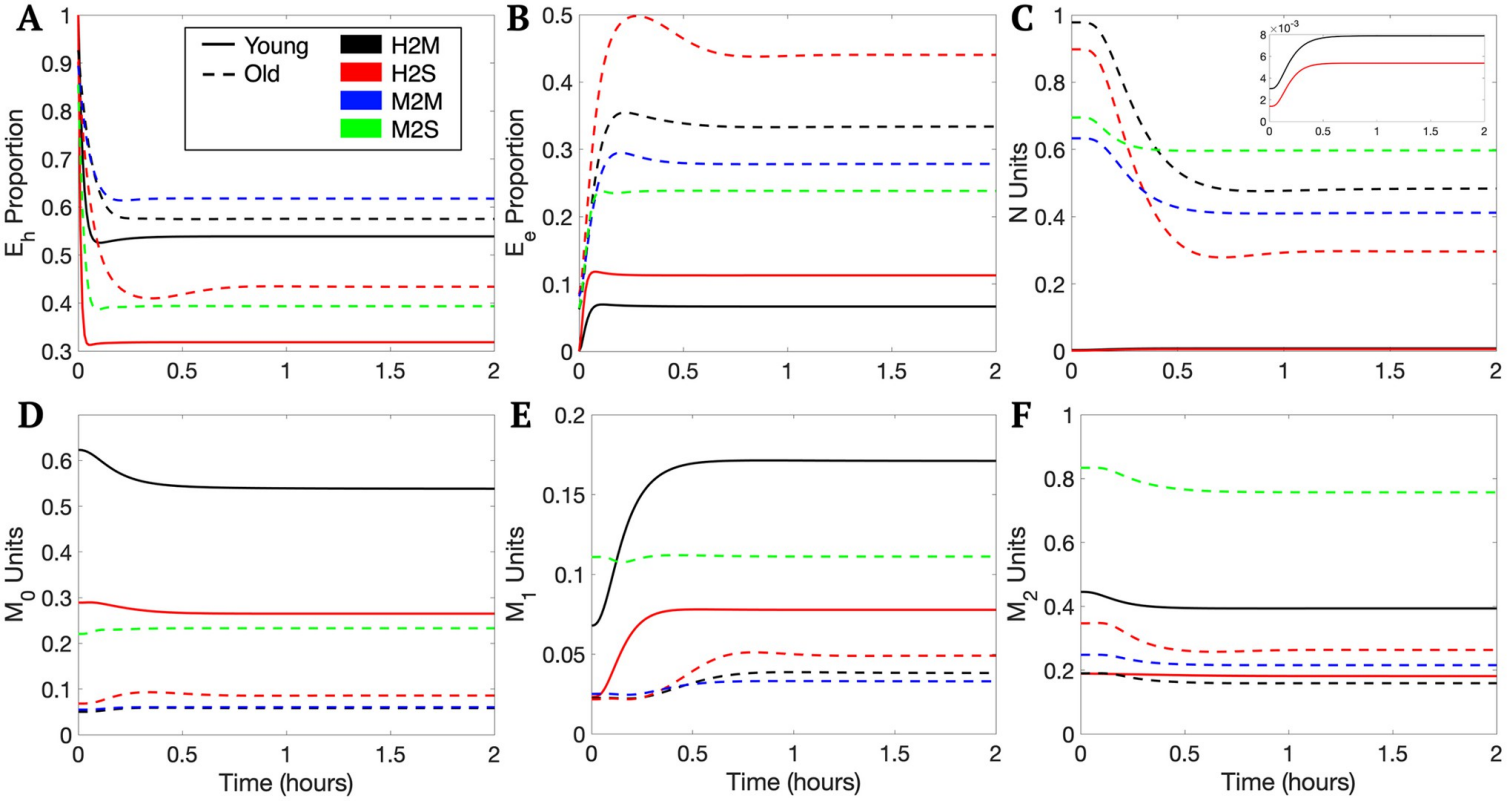
Plots of selected model variables for each representative set. Transients are plotted for *E*_*h*_ (plot A), *E*_*e*_ (plot B), *N* (plot C), *M*_0_ (plot D), *M*_1_ (plot E), and *M*_2_ (plot F). Due to the differences in scale, plot C also includes a zoomed in plot of just the young transients for the variable *N*.

A representative set was determined for each age and ventilation response group such that it was one of the parameter sets in the group and its behavior was most inline with the mean of the key variable transients for all the parameter sets in its group. The mean of the transients was calculated for each group using the average values at each time point for the model variables *E*_*h*_, *E*_*d*_, *E*_*e*_, *M*_0_, *M*_1_, *M*_2_, *N*, and *AN*. A representative set from each group was then chosen by minimizing the residual sum of squares for all six mean transients. The representative sets are plotted for each variable in Fig 5.

#### Importance factors for age classification

**Fig 6:**
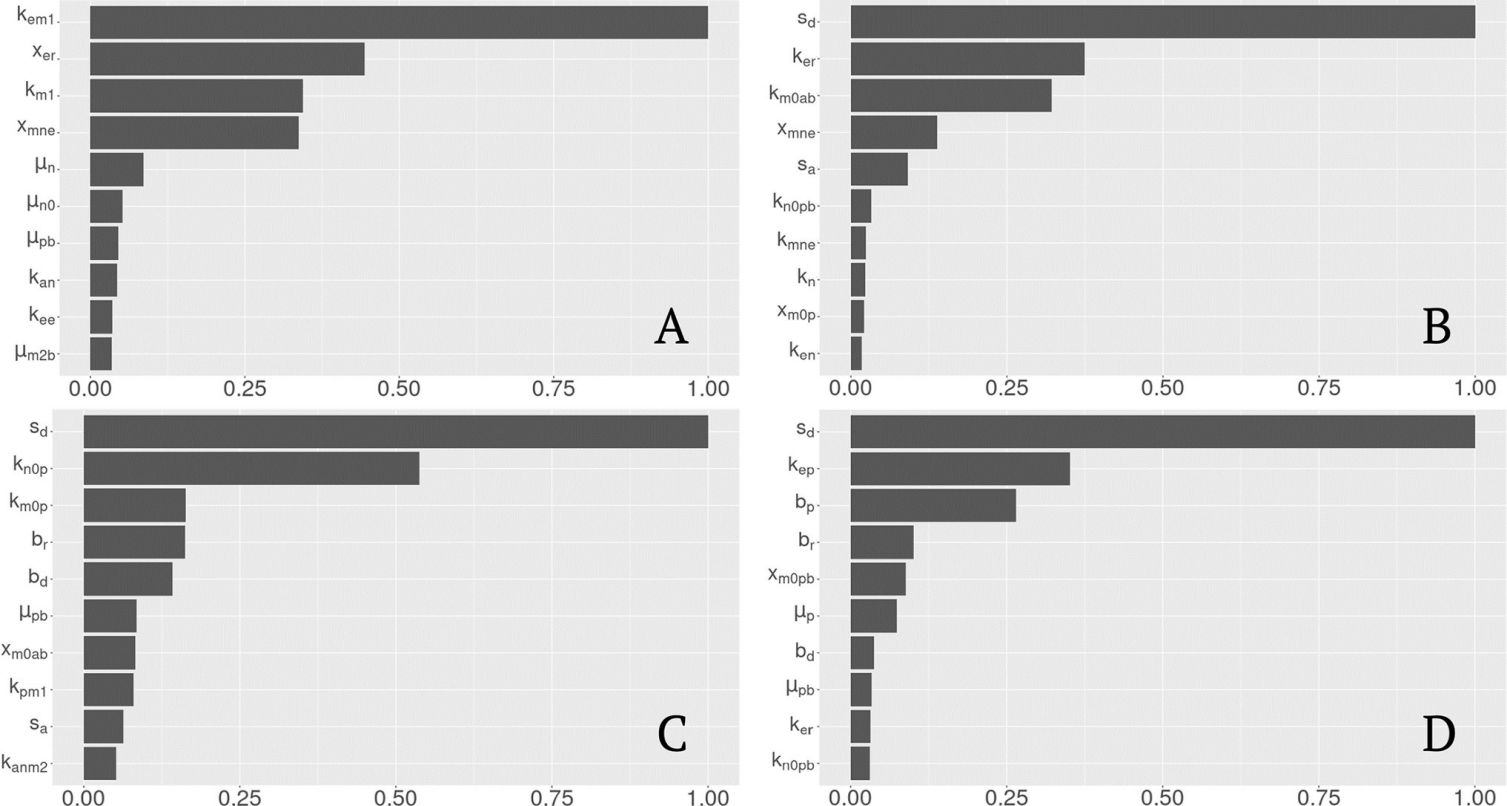
Scaled variable importance for top 10 predictors. Plots A-D exhibit scaled importance values for all observations (predicting young or old), the old class (predicting healthy or moderate at time 0), the healthy, young class (predicting moderate or severe after 2 hours), and the healthy, old class (predicting moderate or severe after 2 hours), respectively.

Importance values for the various classification methods are shown in Fig 6. To account for the difference in sample size between the young and old associated parameter sets, classification methods were performed on random subsets of the parameter sets associated with the young data. For each classification method, down-sampled data yielded similar results; the ordering of parameters often varied, but the parameters most important for each classification remained unchanged. Plot A exhibits ranked parameters in the classification of old and young parameter sets. In terms of scaled importance, the first four parameters are of interest since the numerical value decays significantly after the fourth parameter. Thus, the following parameters had high importance in classifying between the young and old parameter sets: *k*_*em*1_, the rate of phagocytosis of damaged cells by *M*_1_, *x*_*er*_, a Hill-type constant regulating the effectiveness of repair of damaged cells by repair mediators, *k*_*m*1_, the rate of *M*_1_ infiltration into the alveolar compartment, which is novel to this iteration of the model, and *x*_*mne*_, a Hill-type constant regulating the effectiveness of macrophages and neutrophils to damage epithelial cells.

The parameters *k*_*em*1_, *x*_*er*_, and *x*_*mne*_ specifically involve repair and damage of the epithelial cells. The experimental data (Fig 2, “Airspace Enlargment”) and *in silico* simulations (Fig 3, “*E*_*e*_”) both exhibited a discrepancy in the epithelial variables. In terms of the actual data, the experimental range for airspace enlargement (Fig 2, “Airspace Enlargement”, correlated with the model variable *E*_*e*_), does overlap between young and old mice, but a general increase in this value is observed in the old mice. However, simulations show a clear distinction in the variable *E*_*e*_ (Fig 3, “*E*_*e*_”). Therefore, the classification methods found parameters associated with epithelial dynamics important in separating parameter sets between the two age groups. Why other parameters affecting this variable were not found to be as important is unclear.

The parameters *k*_*m*1_, *k*_*em*1_, and *x*_*mne*_ involve macrophages, specifically the M1 phenotype. This was not the expected phenotype to be associated with age classification given that the experimental groups have non-overlapping ranges for M0% and M2% (Fig 2) and for the *in silico* simulations (Fig 3). However, the M1 phenotype changes are driven by the ventilation and their percentages are directly linked to the M0 and M2, thus activation of the M1 phenotype directly affects population numbers of the M0 and M2 phenotypes.

An additional classification suite was performed for the old parameter sets prior to ventilation, shown in Fig 6B, since there was variety in the starting states of these simulations. The parameter *s*_*d*_ holds the largest importance value as it contributes directly to damage of the epithelial cells by the ventilator. The parameters *k*_*er*_, the rate of repair to damaged cells by repair mediators, and *x*_*mne*_, a Hill-type constant regulating the effectiveness of macrophages and neutrophils to damage epithelial cells, are both parameters in the epithelial equations contributing to damage and repair processes. The parameters *k*_*m*0*ab*_, the rate of differentiation of *M*_0*b*_ by *a*_*b*_, and *s*_*a*_, the source rate of background *a*_*b*_, also had high importance factors. Both parameters are involved in the function of AIMs that have not yet been discussed in the topic of lung health classification. AIMs as well as their cellular counterparts, namely M2 macrophages, help regulate the pro-inflammatory response and are crucial to preventing a feedback loop of chronic inflammation. As discussed earlier, both the pro- and anti-inflammatory responses are needed to ensure effective healing. Thus, despite their absence in the epithelial equations, AIMs are crucial to controlling cellular damage and promoting repair. The classification results reflect this relationship.

#### Importance factors for response

Fig 6C exhibits classification for a moderate or severe state after two hours of ventilation for young parameter sets. The most important parameters in classifying moderate or severe lung health were *s*_*d*_, the rate of epithelial damage from the ventilator, *k*_*n*0*p*_, the rate of activation of *N* in the alveolar compartment, novel to this iteration of the model, *k*_*m*0*p*_, the rate of differentiation of *M*_0_ by *p*, *b*_*r*_, baseline repair of damaged cells, and *b*_*d*_, baseline death rate for damaged cells.

For old sets that started healthy, classification methods were used to determine importance factors for a moderate versus severe response to ventilation, Fig 6D. The most important parameters for classification were *s*_*d*_, *k*_*ep*_, the rate of self-resolving repair mediated by *p*, *b*_*p*_, baseline self-resolving repair of epithelial cells, *b*_*r*_, and *x*_*m*0*pb*_, a Hill-type constant regulating the effectiveness of differentiation of *M*_0*b*_ by *p*_*b*_.

The actual parameters with high importance factors differ between young and old parameter sets but generally involve rates of damage and repair. The parameter *s*_*d*_ was the top parameter for each group. This parameter contributes directly to decreasing *E*_*h*_ and thus influences the resulting classification of lung health. The parameters *b*_*r*_, *b*_*d*_, *k*_*ep*_, and *b*_*p*_ all contribute directly to the epithelial variables and thus influence the level of *E*_*h*_ as well. The remaining parameters *k*_*n*0*p*_, *k*_*m*0*p*_, and *x*_*m*0*pb*_ are not directly involved in the epithelial variables but do contribute indirectly to increased damage. All are involved in activation of inflammatory cells by PIMs. The pro-inflammatory phagocytic cells contribute to additional cellular damage. This is a well-established phenomenon of the inflammatory stage [55, 56].

#### Local sensitivity analysis for representative sets

**Fig 7:**
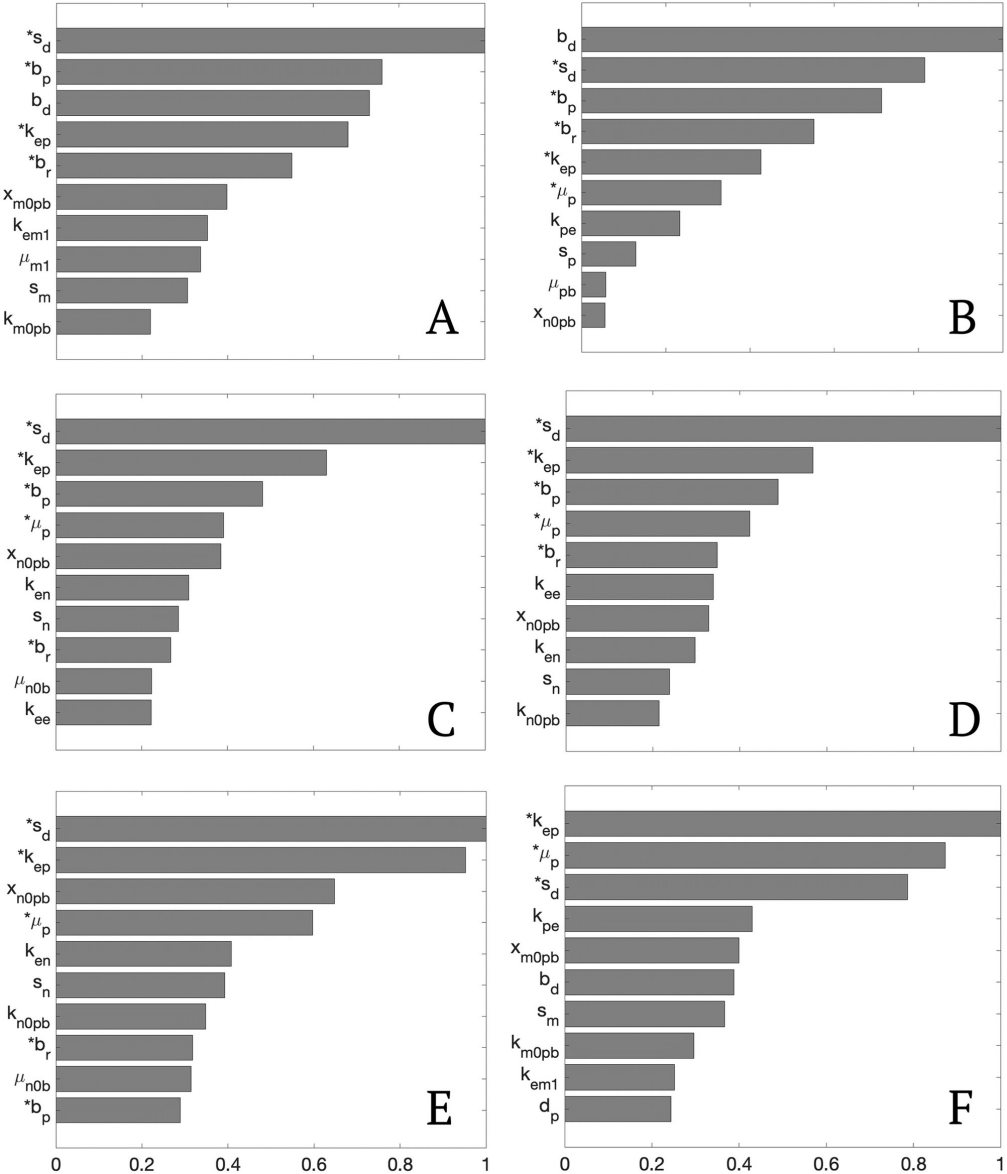
Normalized local sensitivity values for the top ten parameters in each representative set. Plots A-F are the sensitivities for the parameter sets grouped in Young H2M, Young H2S, Old H2M, Old H2S, Old M2M, and Old M2S, respectively. Parameters indicated with an asterisk had sensitivities greater than 10% of the maximum sensitivity value for all representative sets.

Local sensitivity analysis was performed to measure the sensitivity of the variables *E*_*h*_ and *E*_*e*_ to the model parameters. For each of the representative sets a local sensitivity analysis was performed and the top ten sensitive parameters for each representative set are plotted with their normalized value in Fig 7.

The model variables were considered to be sensitive to a parameter if the sensitivity value was larger than 10% of the maximum value for each group. Multiple parameters were identified above this threshold for all of the representative sets, namely; *b*_*p*_, baseline self-resolving repair of epithelial cells, *s*_*d*_, the rate of damage from the ventilator, *b*_*r*_, baseline repair of damaged cells, *k*_*ep*_, the rate of self-resolving repair mediated by *p*, and *μ*_*p*_, decay rate of *p*. These parameters mainly affect accumulation of damage, ability to repair, and dynamics involving PIMs. Since *E*_*h*_ and *E*_*e*_ were the variables used in the sensitivity calculations, it is unsurprising that the parameters in those equations had high sensitivity values. We also again see parameters involved in the pro-inflammatory response to be important.

Additional parameters were found to be sensitive in the majority of the groups. Parameters *s*_*p*_, source of PIMs, *s*_*n*_, source of neutrophils, and *k*_*en*_, rate of phagocytosis of damaged cells by neutrophils, had sensitivity values larger than 10% of the maximum in all groups except Young H2M. The parameter *x*_*n*0*pb*_, a Hill-type parameter involved in the activation of neutrophils by PIM, had a sensitivity value larger than 10% of the maximum in all the old groups. Again, we see parameters involved in PIMs and inflammatory cell activation as well as their processes. Interestingly, the parameter *k*_*em*1_, phagocytosis of damaged cells by M1 macrophages, had small sensitivity values for the majority of the groups. This is interesting since it was the top parameter identified in separating parameter sets associated with the young or old data. However, while the parameter values clearly differ between the parameter sets associated with either the young or old data, the parameter itself does not appear to have a major affect on the proportion of healthy or dead epithelial cells in the lung tissue. This is consistent with previous research which has hypothesized that a sustained inflammatory insult is primarily mediated by neutrophils rather than macrophages, contributing to the development of acute lung injury and ARDS [57].

#### Modulating response to ventilation

The results of the local sensitivity analysis were used to simulate a pseudo-intervention for the parameter sets associated with a severe state after 2 hours of ventilation; Young H2S, Old H2S, and Old M2S.

The parameters chosen as influential across all groups from the sensitivity analysis were *b*_*p*_, *s*_*d*_, *b*_*r*_, *k*_*ep*_, and *μ*_*p*_. These parameters were increased or decreased by 10% one at a time and the percent increase or decrease in the variables *E*_*h*_ and *E*_*e*_ at 2 hours was calculated for each parameters set within the Young H2S, Old H2S, and Old M2S groups. Table 3 shows the minimum, mean, and maximum percent change for each variable for each of these three groups. The supplementary materials include this same procedure with the additional parameters identified as influential in the majority of the groups as well as an intervention starting after one hour of ventilation rather than prior to ventilation as shown here.

**Table 3:** Variable effects of modulating parameters.

Parameter		Young H2S						Old H2S						Old M2S					
		Min.		Mean		Max.		Min.		Mean		Max.		Min.		Mean		Max.	
		*E*_*h*_	*E*_*e*_	*E*_*h*_	*E*_*e*_	*E*_*h*_	*E*_*e*_	*E*_*h*_	*E*_*e*_	*E*_*h*_	*E*_*e*_	*E*_*h*_	*E*_*e*_	*E*_*h*_	*E*_*e*_	*E*_*h*_	*E*_*e*_	*E*_*h*_	*E*_*e*_
*b*_*d*_	-	-2.21%	-2.46%	-0.48%	-0.82%	0.51%	0.00%	-1.50%	-5.73%	0.29%	-1.15%	2.45%	0.00%	-2.91%	-41.25%	0.64%	-4.47%	17.80%	-0.02%
	+	-0.52%	0.00%	0.44%	0.81%	2.00%	2.53%	-2.93%	0.00%	-0.30%	1.13%	1.40%	5.24%	-15.35%	0.02%	-0.60%	3.98%	2.65%	40.92%
*b_p_*	-	-3.30%	0.17%	-0.67%	5.22%	-0.01%	10.93%	-11.65%	0.03%	-3.25%	3.86%	-0.02%	11.81%	-21.27%	0.29%	-3.96%	5.15%	-0.07%	46.91%
	+	0.01%	-8.96%	0.59%	-4.60%	2.72%	-0.17%	0.02%	-9.31%	2.87%	-3.37%	8.86%	-0.03%	0.07%	-39.85%	3.70%	-4.81%	19.95%	-0.29%
*s*_*d*_	-	4.44%	-8.16%	6.94%	-3.27%	10.31%	-0.37%	4.29%	-12.33%	6.93%	-4.90%	12.03%	-1.83%	3.94%	-42.01%	8.36%	-5.00%	28.36%	-1.84%
	+	-8.55%	0.31%	-6.07%	2.88%	-4.04%	7.59%	-12.24%	1.56%	-6.48%	4.81%	-3.97%	16.07%	-22.60%	1.60%	-7.16%	4.29%	-3.65%	41.45%
*b*_*r*_	-	-6.78%	0.00%	-3.60%	1.93%	0.00%	7.49%	-5.93%	0.02%	-2.56%	1.84%	-0.08%	4.98%	-5.27%	0.05%	-1.90%	1.06%	-0.13%	4.05%
	+	0.00%	-6.73%	3.44%	-1.83%	6.66%	0.00%	0.08%	-4.56%	2.41%	-1.71%	5.35%	-0.02%	0.13%	-4.10%	1.83%	-1.03%	5.08%	-0.05%
*k*_*ep*_	-	-3.81%	0.21%	-0.68%	5.73%	-0.04%	12.03%	-13.19%	0.07%	-3.17%	4.14%	-0.06%	13.78%	-18.39%	0.09%	-3.04%	5.03%	-0.09%	40.39%
	+	0.04%	-9.67%	0.60%	-5.04%	3.08%	-0.21%	0.06%	-9.90%	2.77%	-3.59%	9.27%	-0.07%	0.09%	-41.48%	2.98%	-4.82%	22.39%	-0.09%
*k*_*pe*_	-	-2.76%	-8.22%	-0.53%	2.45%	0.95%	9.18%	-1.99%	-0.60%	-0.52%	0.67%	0.39%	4.00%	-1.38%	-0.76%	-0.40%	0.90%	0.41%	4.32%
	+	-0.92%	-7.49%	0.51%	-2.22%	2.46%	8.12%	-0.41%	-3.81%	0.51%	-0.65%	1.96%	0.63%	-0.40%	-4.13%	0.39%	-0.88%	1.34%	0.74%
*μ*_*p*_	-	-1.57%	-9.07%	0.50%	-1.95%	2.90%	11.21%	-2.45%	-8.81%	2.29%	-2.54%	7.69%	3.66%	-1.34%	-46.37%	3.16%	-4.61%	26.23%	2.11%
	+	-2.40%	-9.14%	-0.45%	1.87%	1.25%	9.11%	-7.64%	-2.29%	-2.14%	2.43%	1.42%	8.81%	-18.99%	-1.79%	-2.53%	3.81%	1.12%	41.28%

Minimum, mean, and maximum change in the variables *E*_*h*_ and *E*_*e*_ from a 10% decrease (indicated by “-”) or a 10% increase (indicated by “+”) in the listed parameters. Values are shaded on a sliding scale where darker colors represent numbers with a larger magnitude and lighter colors represent numbers with a smaller magnitude. Maximum and minimum values for color gradient were defined separately for the minimum, mean, and maximum columns. For the minimum in each group, induced decreases in the value of *E*_*h*_ and *E*_*e*_ are shades of orange and induced increases in the value of *E*_*h*_ and *E*_*e*_ are shades of blue.

Parameters had varying effects on the variables, with some increasing *E*_*h*_ while simultaneously increasing *E*_*e*_ as well; thus not necessarily improving health outcome. However, an increase in the parameters *b*_*p*_, *b*_*r*_, and *k*_*ep*_ consistently increased *E*_*h*_ and decreased *E*_*e*_ with a decrease in the parameter producing the opposite effect. The parameter *s*_*d*_ exhibited the inverse relationship where an increase generally produced a decrease in *E*_*h*_ and an increase in *E*_*e*_ with a decrease in this parameter producing the opposite effect. It is also clear by the color intensities that variation in the parameters generally had the most impact on the parameter sets in the Old M2S group. The largest impact was specifically observed in the variable *E*_*e*_. Since only *E*_*h*_ was used to define the possible health states, a large change in *E*_*e*_ may not result in a change in the health state as we have defined it.

**Fig 8:**
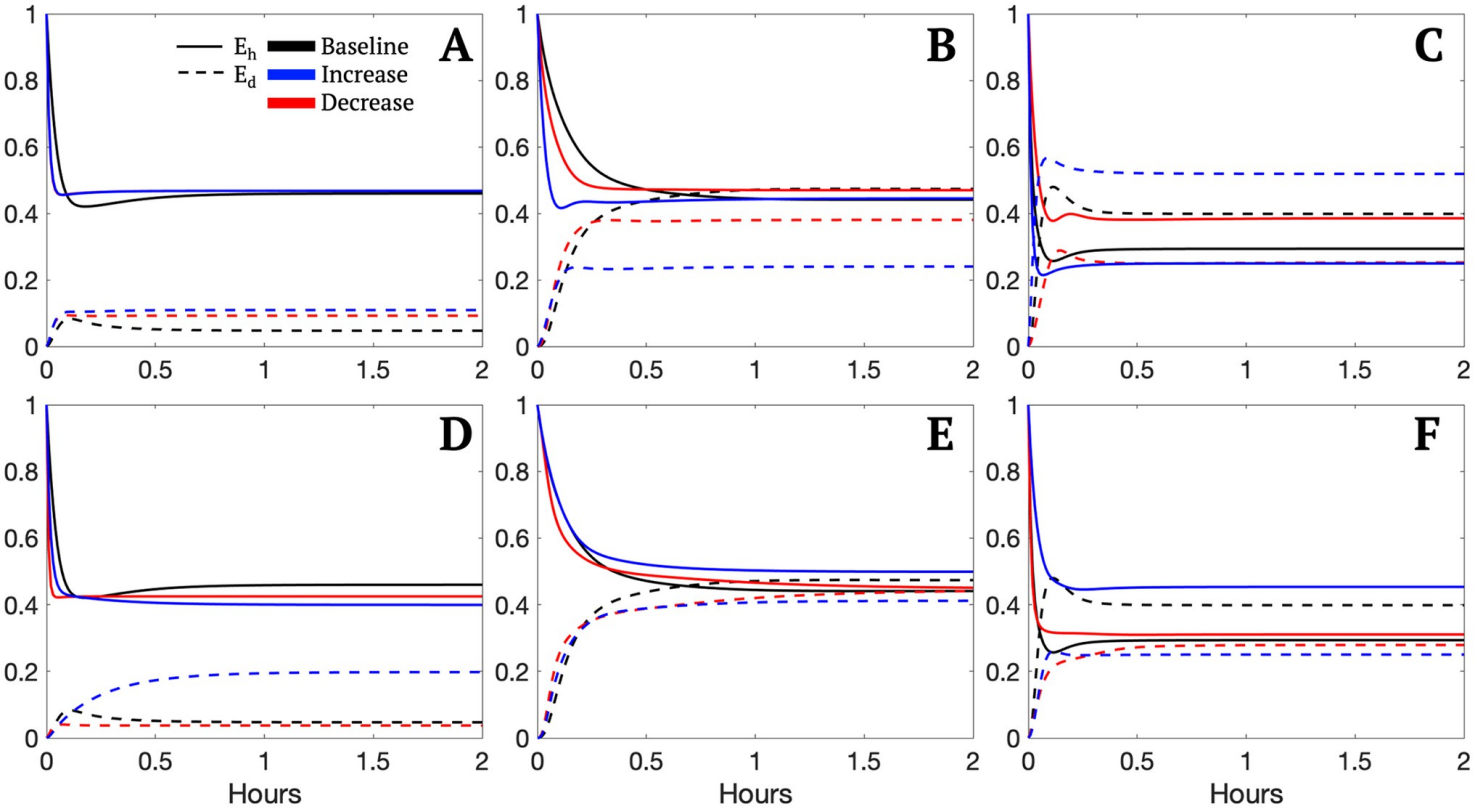
*E*_*h*_ and *E*_*e*_ model transients with varying parameters for example parameter sets from selected groups. Each model variable was simulated for the baseline value, a 10% increase, and a 10% decrease for the selected parameter. Plots A-C exhibit transients for the varied parameter *b*_*r*_ using randomly selected parameter set from the representative groups Young H2S, Old H2S, and Old M2S, respectively. Plots D-F exhibit transients for the varied parameter *s*_*d*_ using a randomly selected parameter set from the representative groups Young H2S, Old H2S, and Old M2S, respectively.

Fig 8 shows some example transients for the variations of the parameters *b*_*r*_ and *s*_*d*_. The variations use the same magnitude shown in Table 3 of a 10% increase or 10% decrease in the selected parameter. The parameter changes appear to have a greater effect on the parameter set associated with the Old M2S representative set (Fig 8C and 8F) which was previously observed in Table 3. Model transients of the variable *E*_*e*_ also show larger differences compared to the transients of *E*_*h*_ as inferred from Table 3. Also note that the general shape of the transients did not change significantly when parameters were varied. Instead the value at which the model variable plateaus changed.

The intent of modulating parameters was to explore potential targets for therapeutic interventions prior to or during MV. The results in Table 3 demonstrate the various outcomes as well as differences among groups. A range of responses is expected in practice since patients have unique health profiles, however, the mean provides a reasonable expectation for the average response. The results shown in Table 3 exhibit the wide range of behavior possible with the model and parameter sets provided and specific groups and parameter combinations produced larger ranges of possible values. Specifically, the Old M2S parameter sets had a high level of variability in the observed maximums, especially for the variable *E*_*e*_. The parameters *b*_*p*_, *k*_*ep*_, *s*_*d*_, and *μ*_*p*_ produced a possible difference of around 40–50% in the variable *E*_*e*_. Potential large decreases were also observed for the same parameters producing a decrease in *E*_*e*_ at similar magnitudes. Generally, overall averages were not as considerable and were all less than 10% in absolute value, with some producing average differences close to zero. This suggests that on average, we would not expect a significant change in the defined health state, unless the simulation was already relatively close to the set threshold. Despite this, even a 2–3% increase or decrease in the amount of healthy epithelial cells available for gas exchange may affect clinical presentation. More research would need to be done to assess this claim.

## Discussion

Age-dependent responses to ventilation are of medical interest given the increased need for ventilation and increased mortality rates of ventilated patients associated with age. Further, despite the clinical need for better understanding of age-related VILI, there is no consensus on VILI models that are used experimentally, and few existing experimental models have tested aged subjects [40]. Using mathematical modeling and statistical methods, we analyzed plausible ventilator responses associated with experimental groups for young and old mice with 2 hours of ventilation using macrophage phenotype and lung integrity data. Our mathematical model calibrated with data from one commonly used mouse VILI model, high-pressure mechanical ventilation for 2 hours, may be used to test future VILI models and plan experiments with clinically meaningful results.

The experimental data was used as acceptance criteria to identify parameter sets associated with the young or old data. Thus, differences observed in the *in silico* model transients of *M*_0_, *M*_1_, *M*_2_, and *E*_*e*_ match those observed in the experimental data. Neutrophil counts, however, were not included in data collection, but do exhibit an observable difference in the model simulations as seen in Figs 3 and 5. The old transients exhibited much higher levels of neutrophils compared with the young sets. This is consistent with *in vivo* results where increased neutrophil infiltration and alveolar damage were correlated [58]. Higher neutrophil infiltration has also been observed in older individuals following lung injury [59].

Classification results revealed that parameters involved in repair and damage of epithelial cells were important in separating parameter sets into the young or old experimental groups. The parameters involved in repair and damage of epithelial cells were expected results given the discrepancies observed in the airspace enlargement variable of the experimental data as well as past research that has shown poorer lung health in aged subjects. The potential for repair and damage in the epithelial tissue offers some insight into what may be driving major differences in the response to ventilation for young and old patients. Parameters involved in macrophage function were also ranked highly in separating parameter sets between the two age groups. These were expected to be associated with differences in parameter sets associated with the young and old data based on past research as well as our experimental findings. Classification results highlighted macrophage parameters specifically relating to the M1 phenotype which was not observed to have discernible differences in the experimental data. However, changes in activation of the M1 phenotype directly affect the populations of the M0 and M2 phenotypes which did have significantly different ranges in the experimental data at baseline. Based on the data, differences are observed in the M0 and M2 phenotype populations at baseline but the classification results indicate that this difference may be related to underlying M1 dynamics.

Classification results based on lung health state identified parameters involved in activation of inflammatory cells and mediators, and parameters involved in damage and repair to be important. These results are expected from the model since repair and damage directly contribute to the variables used to classify the health state. Additionally, the processes involved in the pro-inflammatory response are known to affect local tissue health. Local sensitivity results identified similar parameters involved in damage and repair as well as parameters directly related to the pro-inflammatory response, namely PIMs and neutrophils. Past research has identified a pro-inflammatory response, mainly mediated by neutrophils, to be significant in the development of acute lung injury and ARDS. Our model suggests that neutrophils likely play a role in age-related differences as well since increased neutrophil activation was observed in the parameter sets associated with the old data as well as generally correlating with poorer epithelial health.

To explore how targeted interventions could change the poor responses to ventilation we modulated parameters that model outputs were sensitive to and evaluated changes in the model-predicted epithelial cell health. The local sensitivity results were used to select parameters to modulate and simulations were performed for all parameter sets in each of the representative groups with a severe health state after ventilation. In some cases, a wide range of responses were observed. The greatest effects were observed in the old representative sets, specifically for the variable *E*_*e*_. For the old representative set classified as moderate before ventilation and severe after ventilation, some parameter modulations produced a potential increase or decrease in *E*_*e*_ of about 40–50%. Overall, the mean response to modulation of each parameter had a magnitude less than 10%, with some near 0. Despite this, the targeted parameters offer potentially large improvements, particularly in the old parameter sets with poorer lung health. Additionally, an intervention performed mid-way through ventilation also produced potential improvements in the transients but to a lesser extent.

The exploration of age differences in VILI expresses early attempts to create more personalized medical interventions. Despite the observed differences in MV response with age, clinical use and interventions for MV remain a one-size-fits-all approach, ignoring underlying immunological variance in patients. Our model suggests that parameters controlling M1 macrophage dynamics and pro-inflammatory mediators show distinct separation between young and old associated parameter sets. These could be potential targets for further research to help identify the cause of the differing responses to ventilation in young and old subjects. Simulated interventions indicated damage and repair parameters showed the potential to improve tissue health during ventilation, with these being most influential for old associated parameter sets with poorer lung health. However, the wide range of potential responses indicates there are more components involved in alveolar tissue health.

This model can be adapted to account for non-VILI associated lung injury, such as an infection or inhaled toxins. The statistical and mathematical method used with this model can then determine key components of the immune and repair responses for those insults. Insults could be combined with ventilation to better explore the age-dependent response to VILI while accounting for co-factors that lead to the necessity for ventilation.

## Supporting information

S1 Fig Supplementary flow cytometry table. (TIF)

S1 Eq M0 macrophage equations. (PDF)

S2 Eq M1 macrophage equations. (PDF)

S3 Eq M2 macrophage equations. (PDF)

S4 Eq Neutrophil equations. (PDF)

S5 Eq Pro- and anti-inflammatory mediators equations. (PDF)

S6 Eq Repair and epithelial equations. (PDF)

S1 Table Full table modulating parameters prior to ventilation. Minimum, mean, and maximum change in the variables *E*_*h*_ and *E*_*e*_ from a 10% decrease (indicated by “-”) or a 10% increase (indicated by “+”) in the listed parameters. Values are shaded on a sliding scale where darker colors represent numbers with a larger magnitude and lighter colors represent numbers with a smaller magnitude. For the minimum in each group, induced decreases in the value of *E*_*h*_ and *E*_*e*_ are orange and induced increases in the value of *E*_*h*_ and *E*_*e*_ are blue.
(TIF)

S2 Table Full table modulating parameters after 1 hour of ventilation. Minimum, mean, and maximum change in the variables *E*_*h*_ and *E*_*e*_ from a 10% decrease (indicated by “-”) or a 10% increase (indicated by “+”) in the listed parameters after 1 hour of ventilation. Values are shaded on a sliding scale where darker colors represent numbers with a larger magnitude and lighter colors represent numbers with a smaller magnitude. For the minimum in each group, induced decreases in the value of *E*_*h*_ and *E*_*e*_ are orange and induced increases in the value of *E*_*h*_ and *E*_*e*_ are blue.
(TIF)

## References

[1] Williams GW, Berg NK, Reskallah A, Yuan X, Eltzschig HK. Acute Respiratory Distress Syndrome: Contemporary Management and Novel Approaches during COVID-19. Anesthesiology. 2021;134(2):270–282. doi: 10.1097/ALN.0000000000003571 33016981 PMC7854846

[2] Halbertsma FJJ, Vaneker M, Scheffer GJ, van der Hoeven JG. Cytokines and biotrauma in ventilator-induced lung injury: a critical review of the literature. The Netherlands Journal of Medicine. 2005;63(10):382–392. 16301759

[3] Slutsky AS, Ranieri VM. Ventilator-Induced Lung Injury. New England Journal of Medicine. 2013;369(22):2126–2136. doi: 10.1056/NEJMra1208707 24283226

[4] Provinciali M, Cardelli M, Marchegiani F. Inflammation, chronic obstructive pulmonary disease and aging. Current Opinion in Pulmonary Medicine. 2011;17:S3. doi: 10.1097/01.mcp.0000410742.90463.1f 22209928

[5] Rekeneire Nd, Peila R, Ding J, Colbert LH, Visser M, Shorr RI, et al. Diabetes, Hyperglycemia, and Inflammation in Older Individuals: The Health, Aging and Body Composition study. Diabetes Care. 2006;29(8):1902–1908. doi: 10.2337/dc05-2327 16873800

[6] Poletti P, Tirani M, Cereda D, Trentini F, Guzzetta G, Sabatino G, et al. Association of Age With Likelihood of Developing Symptoms and Critical Disease Among Close Contacts Exposed to Patients With Confirmed SARS-CoV-2 Infection in Italy. JAMA Network Open. 2021;4(3):e211085–e211085. doi: 10.1001/jamanetworkopen.2021.1085 33688964 PMC7948061

[7] Setzer F, Oschatz K, Hueter L, Schmidt B, Schwarzkopf K, Schreiber T. Susceptibility to ventilator induced lung injury is increased in senescent rats. Critical Care. 2013;17:R99. doi: 10.1186/cc12744 23710684 PMC4056597

[8] Herbert JA, Valentine MS, Saravanan N, Schneck MB, Pidaparti R, Fowler AA, et al. Conservative fluid management prevents age-associated ventilator induced mortality. Experimental Gerontology. 2016;81:101–109. doi: 10.1016/j.exger.2016.05.005 27188767 PMC5589142

[9] Bruno G, Perelli S, Fabrizio C, Buccoliero GB. Short-term outcomes in individuals aged 75 or older with severe coronavirus disease (COVID-19): First observations from an Infectious Diseases Unit in Southern Italy. The Journal of Infection. 2020. doi: 10.1016/j.jinf.2020.05.024 32417315 PMC7224683

[10] Canan CH, Gokhale NS, Carruthers B, Lafuse WP, Schlesinger LS, Torrelles JB, et al. Characterization of Lung Inflammation and its Impact on Macrophage Function in Aging. Journal of Leukocyte Biology. 2014;96(3):473–480. doi: 10.1189/jlb.4A0214-093RR 24935957 PMC4632167

[11] Nin N, Lorente JA, De Paula M, Fernández-Segoviano P, Peñuelas O, Sánchez-Ferrer A, et al. Aging increases the susceptibility to injurious mechanical ventilation. Intensive Care Medicine. 2008;34. doi: 10.1007/s00134-007-0960-0 18180905

[12] Panda A, Arjona A, Sapey E, Bai F, Fikrig E, Montgomery RR, et al. Human innate immunosenescence: causes and consequences for immunity in old age. Trends Immunol. 2009;30. doi: 10.1016/j.it.2009.05.004 19541535 PMC4067971

[13] Mahbub S, Brubaker AL, Kovacs EJ. Aging of the Innate Immune System: An Update. Curr Immunol Rev. 2011;7. doi: 10.2174/157339511794474181 21461315 PMC3066013

[14] Arnold CR, Wolf J, Brunner S, Herndler-Brandstetter D, Grubeck-Loebenstein B. Gain and Loss of T Cell Subsets in Old Age—Age-Related Reshaping of the T Cell Repertoire. Journal of Clinical Immunology. 2011;31. doi: 10.1007/s10875-010-9499-x 21243520

[15] Dace DSARS. Effect of senescence on macrophage polarization and angiogenesis. Rejuvenation research. 2008;11:177–185. doi: 10.1089/rej.2007.0614 18279031

[16] Linehan E, Fitzgerald D. Ageing and the Immune System: Focus on Macrophages. European Journal of Microbiology and Immunology. 2015;5(1):14–24. doi: 10.1556/EUJMI-D-14-00035 25883791 PMC4397845

[17] Mahbub S, Deburghgraeve CR, Kovacs EJ. Advanced Age Impairs Macrophage Polarization. Journal of Interferon & Cytokine Research. 2011;32(1):18–26. doi: 10.1089/jir.2011.0058 22175541 PMC3255514

[18] Matthay MA, Robriquet L, Fang X. Alveolar Epithelium. Proceedings of the American Thoracic Society. 2005;2(3):206–213. doi: 10.1513/pats.200501-009AC 16222039

[19] Mason RJ. Biology of alveolar type II cells. Respirology. 2006;11(s1):S12–S15. doi: 10.1111/j.1440-1843.2006.00800.x 16423262

[20] Eberhardt M, Lai X, Tomar N, Gupta S, Schmeck B, Steinkasserer A, et al. Third-Kind Encounters in Biomedicine: Immunology Meets Mathematics and Informatics to Become Quantitative and Predictive. Methods in Molecular Biology (Clifton, NJ). 2016;1386:135–179. doi: 10.1007/978-1-4939-3283-2_9 26677184

[21] Minucci S, Heise RL, Valentine MS, Kamga Gninzeko FJ, Reynolds AM. Mathematical modeling of ventilator-induced lung inflammation. Journal of Theoretical Biology. 2021;526:110738. doi: 10.1016/j.jtbi.2021.110738 33930440 PMC8277755

[22] An G, Cockrell C, Zamora R, Vodovotz Y. 11—Machine learning and mechanistic computational modeling of inflammation as tools for designing immunomodulatory biomaterials. In: Badylak SF, Elisseeff JH, editors. Immunomodulatory Biomaterials. Woodhead Publishing Series in Biomaterials. Woodhead Publishing; 2021. p. 251–272. Available from: https://www.sciencedirect.com/science/article/pii/B9780128214404000098.

[23] McKay MD, Beckman RJ, Conover WJ. A Comparison of Three Methods for Selecting Values of Input Variables in the Analysis of Output from a Computer Code. Technometrics. 1979;21(2):239–245. doi: 10.1080/00401706.1979.10489755

[24] Johnson ME, Moore LM, Ylvisaker D. Minimax and maximin distance designs. Journal of Statistical Planning and Inference. 1990;26(2):131–148. doi: 10.1016/0378-3758(90)90122-B

[25] Qian P. Sliced Latin Hypercube Designs. Journal of The American Statistical Association. 2012;107. doi: 10.1080/01621459.2011.644132

[26] Lekivetz R, Jones B. Fast Flexible Space-Filling Designs for Nonrectangular Regions. Quality and Reliability Engineering International. 2015;31(5):829–837. doi: 10.1002/qre.1640

[27] Janes KA, Yaffe MB. Data-driven modelling of signal-transduction networks. Nature Reviews Molecular Cell Biology. 2006;7(11):820–828. doi: 10.1038/nrm2041 17057752

[28] Mi Q, Constantine G, Ziraldo C, Solovyev A, Torres A, Namas R, et al. A Dynamic View of Trauma/Hemorrhage-Induced Inflammation in Mice: Principal Drivers and Networks. PLOS ONE. 2011;6(5):1–12. doi: 10.1371/journal.pone.0019424 21573002 PMC3091861

[29] Namas RA, Namas R, Lagoa C, Barclay D, Mi Q, Zamora R, et al. Hemoadsorption Reprograms Inflammation in Experimental Gram-negative Septic Peritonitis: Insights from In Vivo and In Silico Studies. Molecular Medicine. 2012;18(10):1366–1374. doi: 10.2119/molmed.2012.00106 22751621 PMC3533640

[30] Wolf MT, Vodovotz Y, Tottey S, Brown BN, Badylak SF. Predicting In Vivo Responses to Biomaterials via Combined In Vitro and In Silico Analysis. Tissue Engineering Part C: Methods. 2015;21(2):148–159. doi: 10.1089/ten.tec.2014.0167 24980950 PMC4313398

[31] Namas RA, Almahmoud K, Mi Q, Ghuma A, Namas R, Zaaqoq A, et al. Individual-specific principal component analysis of circulating inflammatory mediators predicts early organ dysfunction in trauma patients. Journal of Critical Care. 2016;36:146–153. doi: 10.1016/j.jcrc.2016.07.002 27546764 PMC5097026

[32] Saltelli A, Chan K, Scott E. Wiley series in probability and statistics. Sensitivity analysis. 2000;.

[33] Marino S, Hogue IB, Ray CJ, Kirschner DE. A methodology for performing global uncertainty and sensitivity analysis in systems biology. Journal of Theoretical Biology. 2008;254(1):178–196. doi: 10.1016/j.jtbi.2008.04.011 18572196 PMC2570191

[34] Saltelli A. Making best use of model evaluations to compute sensitivity indices. Computer physics communications. 2002;145(2):280–297. doi: 10.1016/S0010-4655(02)00280-1

[35] Krishna NA, Pennington HM, Coppola CD, Eisenberg MC, Schugart RC. Connecting local and global sensitivities in a mathematical model for wound healing. Bulletin of mathematical biology. 2015;77(12):2294–2324. doi: 10.1007/s11538-015-0123-3 26597096

[36] Marino S, Myers A, Flynn JL, Kirschner DE. TNF and IL-10 are Major Factors in Modulation of the Phagocytic Cell Environment in Lung and Lymph Node in Tuberculosis: a Next Generation Two Compartmental Model. Journal of theoretical biology. 2010;265(4):586–598. doi: 10.1016/j.jtbi.2010.05.012 20510249 PMC3150786

[37] Ziraldo C, Solovyev A, Allegretti A, Krishnan S, Henzel MK, Sowa GA, et al. A computational, tissue-realistic model of pressure ulcer formation in individuals with spinal cord injury. PLoS computational biology. 2015;11(6):e1004309. doi: 10.1371/journal.pcbi.1004309 26111346 PMC4482429

[38] Mathew S, Bartels J, Banerjee I, Vodovotz Y. Global sensitivity analysis of a mathematical model of acute inflammation identifies nonlinear dependence of cumulative tissue damage on host interleukin-6 responses. Journal of Theoretical Biology. 2014;358:132–148. doi: 10.1016/j.jtbi.2014.05.036 24909493 PMC4125477

[39] Wu P, Zhao H. Dynamics of an HIV Infection Model with Two Infection Routes and Evolutionary Competition between Two Viral Strains. Applied Mathematical Modelling. 2020;84:240–264. doi: 10.1016/j.apm.2020.03.040

[40] Joelsson JP, Ingthorsson S, Kricker J, Gudjonsson T, Karason S. Ventilator-induced lung-injury in mouse models: Is there a trap? Laboratory Animal Research. 2021;37:1–11. doi: 10.1186/s42826-021-00108-x 34715943 PMC8554750

[41] Card JW, Carey MA, Bradbury JA, DeGraff LM, Morgan DL, Moorman MP, et al. Gender differences in murine airway responsiveness and lipopolysaccharide-induced inflammation. The Journal of Immunology. 2006;177(1):621–630. doi: 10.4049/jimmunol.177.1.621 16785560 PMC2262913

[42] Roche. Collagenase A; 2021.

[43] Roche. DNaseI; 2021.

[44] Yu YRA, Hotten DF, Malakhau Y, Volker E, Ghio AJ, Noble PW, et al. Flow Cytometric Analysis of Myeloid Cells in Human Blood, Bronchoalveolar Lavage, and Lung Tissues. American Journal of Respiratory Cell and Molecular Biology. 2015;54(1):13–24. doi: 10.1165/rcmb.2015-0146OC PMC4742930 26267148

[45] Valentine M, Weigel C, Gninzeko FK, Tho C, Gräler M, Reynolds A, et al. S1P lyase inhibition prevents lung injury following high pressure-controlled mechanical ventilation in aging mice. Experimental Gerontology. 2023;173:112074. doi: 10.1016/j.exger.2022.112074 36566871 PMC9975034

[46] BD Biosciences. FcBlock; 2021.

[47] Misharin AV, Morales-Nebreda L, Mutlu GM, Budinger GRS, Perlman H. Flow Cytometric Analysis of Macrophages and Dendritic Cell Subsets in the Mouse Lung. American Journal of Respiratory Cell and Molecular Biology. 2013;49(4):503–510. doi: 10.1165/rcmb.2013-0086MA 23672262 PMC3824047

[48] BD Biosciences. BD LSRFortessa X-20 Cell Analyzer; 2021.

[49] BD Biosciences. BD FACSDiva; 2021.

[50] Software DN. FCS Express 5 Version 5.00.0065; 2015.

[51] MATLAB. 9.10.0.1739362 (R2021a) Update 5. Natick, Massachusetts: The MathWorks Inc.; 2021.

[52] R Core Team. R: A Language and Environment for Statistical Computing; 2021. Available from: https://www.R-project.org/.

[53] H2O ai Team. h2o: R Interface for H2O Version 3.36.0.4; 2022. Available from: http://www.h2o.ai.

[54] Martins J, Sturdza P, Alonso J. In: The connection between the complex-step derivative approximation and algorithmic differentiation. American Institute of Aeronautics and Astronautics, Inc.; 2001. Available from: https://arc.aiaa.org/doi/abs/10.2514/6.2001-921.

[55] Goris RJA, te Boekhorst TP, Nuytinck JK, Gimbrère JS. Multiple-organ failure: generalized autodestructive inflammation? Archives of surgery. 1985;120(10):1109–1115. doi: 10.1001/archsurg.1985.01390340007001 4038052

[56] Takala A, Jousela I, JANSSON SE, Olkkola KT, Takkunen O, Orpana A, et al. Markers of systemic inflammation predicting organ failure in community-acquired septic shock. Clinical science. 1999;97(5):529–538. doi: 10.1042/cs0970529 10545303

[57] Ware LB. Pathophysiology of acute lung injury and the acute respiratory distress syndrome. In: Seminars in respiratory and critical care medicine. vol. 27. Thieme Medical Publishers, Inc., 333 Seventh Avenue, New …; 2006. p. 337–349. 10.1055/s-2006-948288 16909368

[58] Imanaka H, Shimaoka M, Matsuura N, Nishimura M, Ohta N, Kiyono H. Ventilator-Induced Lung Injury Is Associated with Neutrophil Infiltration, Macrophage Activation, and TGF-*β*1 mRNA Upregulation in Rat Lungs. Anesthesia & Analgesia. 2001;92:428–436. doi: 10.1213/00000539-200102000-00029 11159246

[59] Kling KM, Lopez-Rodriguez E, Pfarrer C, Mühlfeld C, Brandenberger C. Aging exacerbates acute lung injury-induced changes of the air-blood barrier, lung function, and inflammation in the mouse. American Journal of Physiology-Lung Cellular and Molecular Physiology. 2017;312(1):L1–L12. doi: 10.1152/ajplung.00347.2016 27815259

