# Supplementary figures and images for: Age-dependent ventilator-induced lung injury: Mathematical modeling, experimental data, and statistical analysis
Source: PLoS Comput Biol. 2024 Feb 22;20(2):e1011113. doi: 10.1371/journal.pcbi.1011113 (PMC10914268; doi:10.1371/journal.pcbi.1011113)

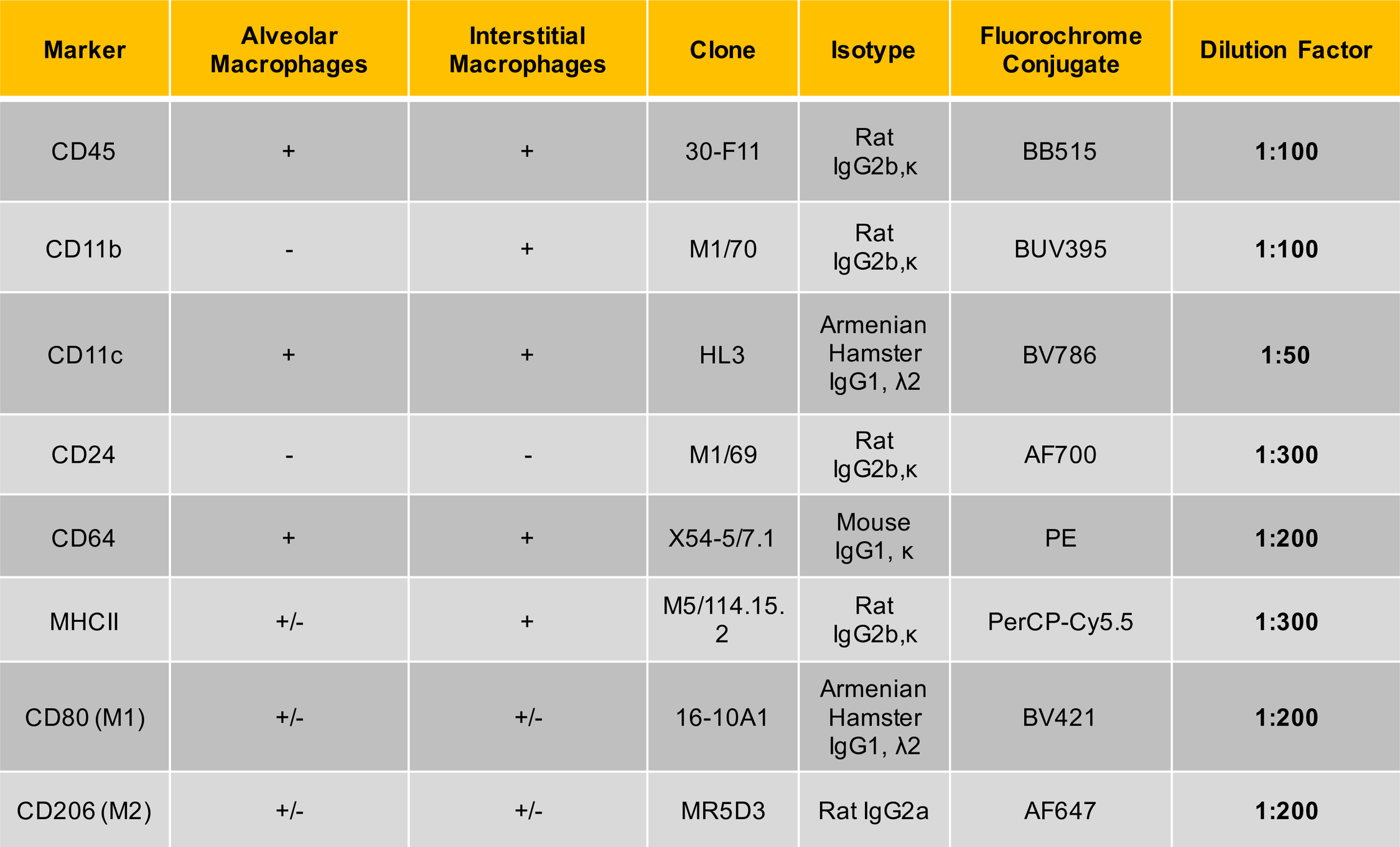

Supplement: S1 Fig — (TIF) [file pcbi.1011113.s001.tif]

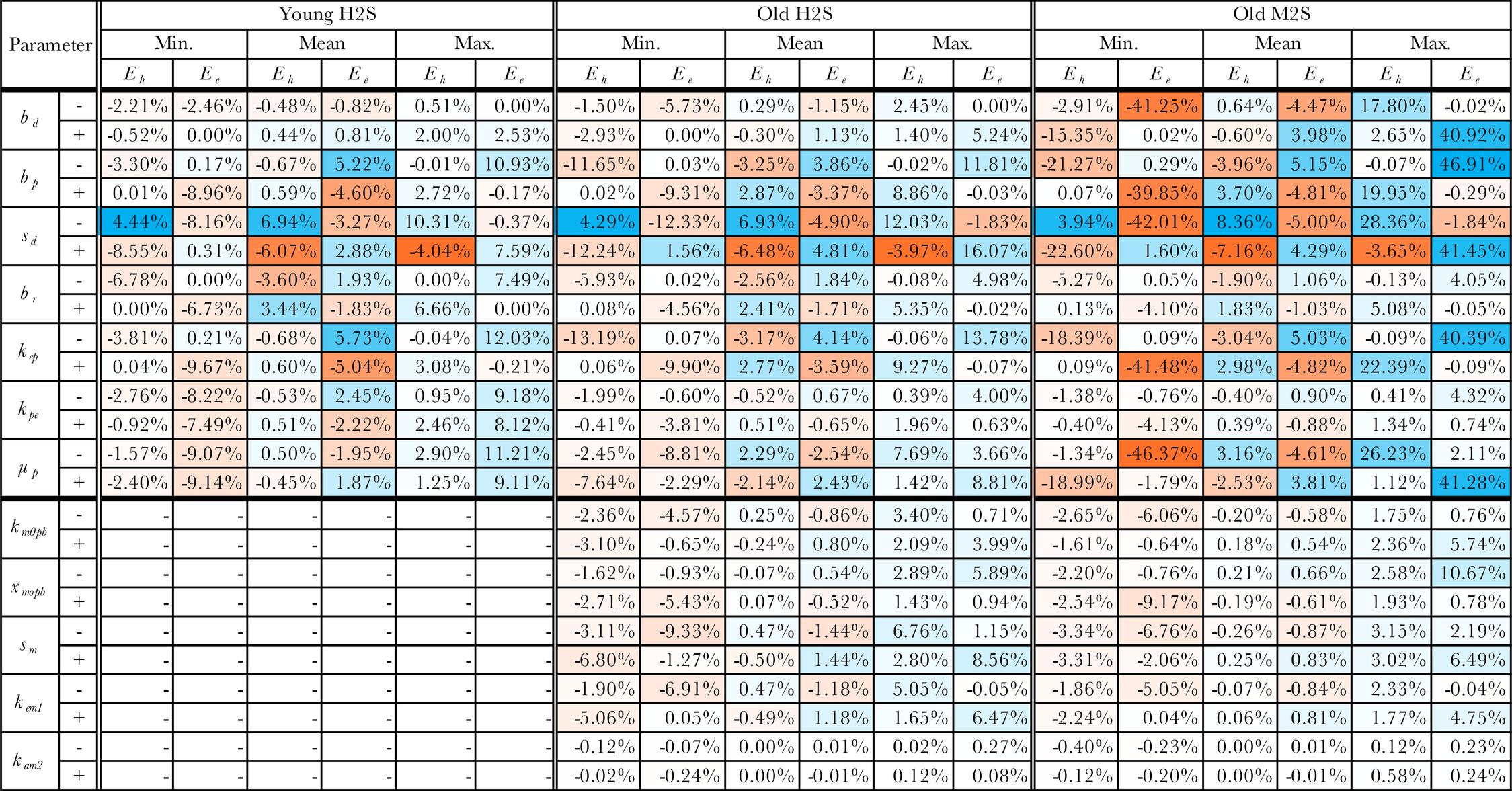

Supplement: S1 Table — Minimum, mean, and maximum change in the variables Eh and Ee from a 10% decrease (indicated by “-”) or a 10% increase (indicated by “+”) in the listed parameters. Values are shaded on a sliding scale where darker colors represent numbers with a larger magnitude and lighter colors represent numbers with a smaller magnitude. For the minimum in each group, induced decreases in the value of Eh and Ee are orange and induced increases in the value of Eh and Ee are blue. (TIF) [file pcbi.1011113.s008.tif]

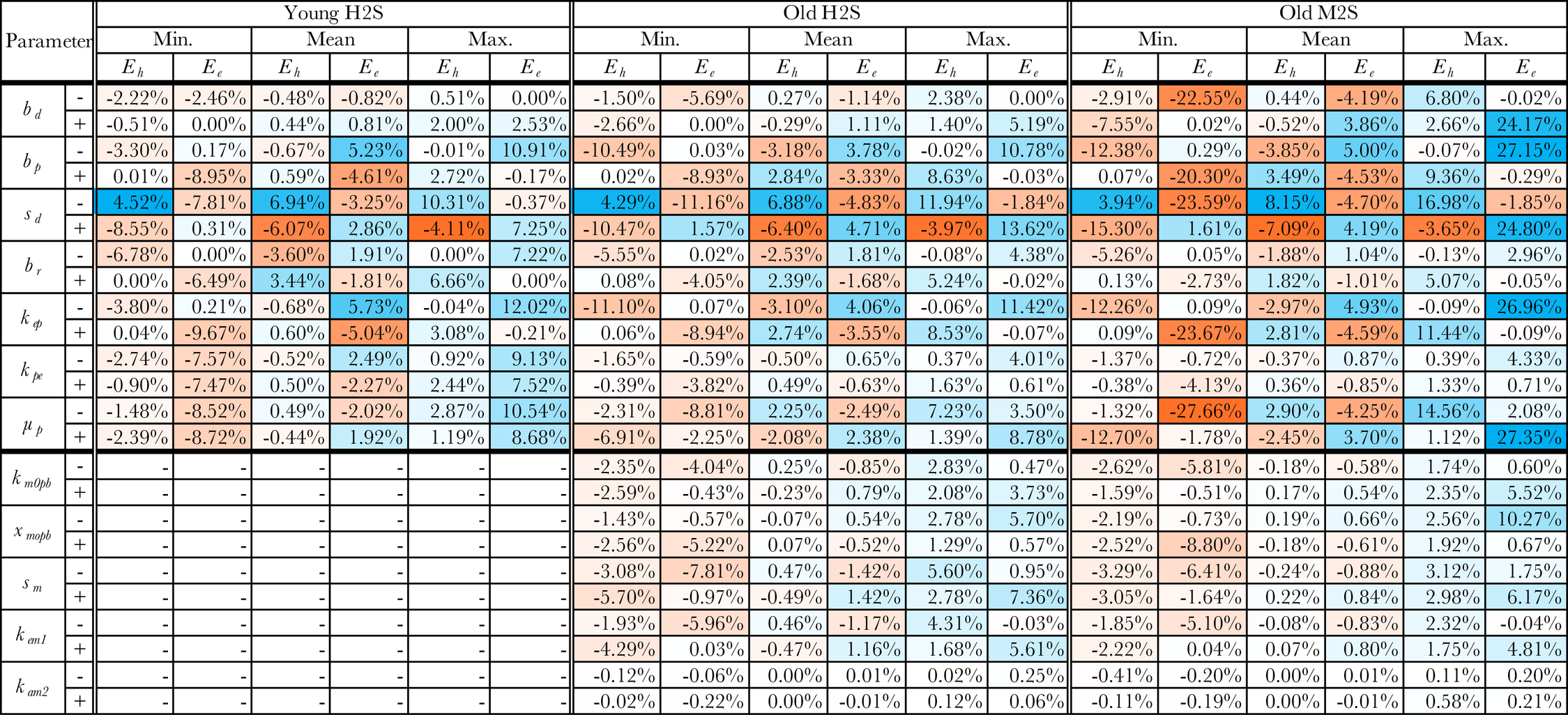

Supplement: S2 Table — Minimum, mean, and maximum change in the variables Eh and Ee from a 10% decrease (indicated by “-”) or a 10% increase (indicated by “+”) in the listed parameters after 1 hour of ventilation. Values are shaded on a sliding scale where darker colors represent numbers with a larger magnitude and lighter colors represent numbers with a smaller magnitude. For the minimum in each group, induced decreases in the value of Eh and Ee are orange and induced increases in the value of Eh and Ee are blue. (TIF) [file pcbi.1011113.s009.tif]
