# Supplementary material for: Age-dependent ventilator-induced lung injury: Mathematical modeling, experimental data, and statistical analysis
Source: PLoS Comput Biol. 2024 Feb 22;20(2):e1011113. doi: 10.1371/journal.pcbi.1011113 (PMC10914268; doi:10.1371/journal.pcbi.1011113)
Supplement: S1 Eq — (PDF) [file pcbi.1011113.s002.pdf]

S1 Eq. M0 macrophage equations

$$\frac{dM_{0b}}{dt} = \underbrace{d_{m0}(M_0 - M_{0b})}_{\text{Diffusion}} + \underbrace{s_m}_{\text{Source}} \underbrace{- M_{0b}\frac{k_{ee}E_e^4}{x_{ee}^4 + E_e^4}}_{\text{Leak into lung}} - \underbrace{\mu_{M0_b} M_{0b}}_{\text{Decay}}$$
$$- \; M_{0b}\left[\underbrace{\left(\frac{k_{m0pb}p_b^2}{x_{m0pb}^2 + p_b^2}\right)}_{\substack{\text{Differentiation} \\ \text{to M1 via PIMs}}}\underbrace{\left(\frac{1}{1 + \left(\frac{a_b}{a_{b\infty}}\right)^2}\right)}_{\substack{\text{Inhibition} \\ \text{by AIMs}}} + \underbrace{\left(\frac{k_{m0ab}a_b^2}{x_{m0ab}^2 + a_b^2}\right)}_{\substack{\text{Differentiation} \\ \text{to M2}}}\right] \tag{1}$$

$$\frac{dM_0}{dt} = -\underbrace{d_{m0}(M_0 - M_{0b})}_{\text{Diffusion}} + \underbrace{M_{0b}\frac{k_{ee}E_e^4}{x_{ee}^4 + E_e^4}}_{\text{Leak into lung}} - \underbrace{\mu_{M_0} M_0}_{\text{Decay}}$$
$$- M_0\left[\underbrace{\left(\frac{k_{m0pb}p_b^2}{x_{m0pb}^2 + p_b^2}\right)}_{\substack{\text{Differentiation} \\ \text{to M1 via PIMs}}}\underbrace{\left(\frac{1}{1 + \left(\frac{a}{a_{\infty}}\right)^2}\right)}_{\substack{\text{Inhibition} \\ \text{by AIMs}}} + \underbrace{\left(\frac{k_{m0a}a^2}{x_{m0a}^2 + a^2}\right)}_{\substack{\text{Differentiation} \\ \text{to M2}}}\right] \tag{2}$$
