# Supplementary material for: Age-dependent ventilator-induced lung injury: Mathematical modeling, experimental data, and statistical analysis
Source: PLoS Comput Biol. 2024 Feb 22;20(2):e1011113. doi: 10.1371/journal.pcbi.1011113 (PMC10914268; doi:10.1371/journal.pcbi.1011113)
Supplement: S2 Eq — (PDF) [file pcbi.1011113.s003.pdf]

S2 Eq. M1 macrophage equations

$$\frac{dM_{1b}}{dt} = M_{0b} \underbrace{\left(\frac{k_{m0pb} p_b^2}{x_{m0pb}^2 + p_b^2} \right)}_{\substack{\text{Differentiation}\\ \text{to M1 via PIMs}}} \underbrace{\left(\frac{1}{1 + \left(\frac{a_b}{a_{b\infty}} \right)^2} \right)}_{\substack{\text{Inhibition}\\ \text{by AIMs}}} - \underbrace{M_{1b} \frac{k_{ee} E_e^4}{x_{ee}^4 + E_e^4}}_{\text{Leak into lung}} - \underbrace{k_{m1} M_{1b}}_{\text{Migration}} - \underbrace{\mu_{M1_b} M_{1b}}_{\text{Decay}} \quad (1)$$

$$\frac{dM_1}{dt} = M_0 \underbrace{\left(\frac{k_{m0p} p^2}{x_{m0p}^2 + p^2} \right)}_{\substack{\text{Differentiation}\\ \text{to M1 via PIMs}}} \underbrace{\left(\frac{1}{1 + \left(\frac{a}{a_\infty} \right)^2} \right)}_{\substack{\text{Inhibition}\\ \text{by AIMs}}} - \underbrace{k_{man}(k_{anm1} A N M_1)}_{\substack{\text{M1 switch to M2}\\ \text{by phagocytosis}}} \underbrace{\left(\frac{1}{1 + \left(\frac{a}{a_\infty} \right)^2} \right)}_{\substack{\text{Inhibition}\\ \text{by AIMs}}}$$

$$+ \underbrace{M_{1b} \frac{k_{ee} E_e^4}{x_{ee}^4 + E_e^4}}_{\text{Leak into lung}} + \underbrace{k_{m1} M_{1b}}_{\text{Migration}} - \underbrace{\mu_{M_1} M_1}_{\text{Decay}} \quad (2)$$
