# Supplementary material for: Age-dependent ventilator-induced lung injury: Mathematical modeling, experimental data, and statistical analysis
Source: PLoS Comput Biol. 2024 Feb 22;20(2):e1011113. doi: 10.1371/journal.pcbi.1011113 (PMC10914268; doi:10.1371/journal.pcbi.1011113)
Supplement: S3 Eq — (PDF) [file pcbi.1011113.s004.pdf]

S3 Eq. M2 macrophage equations

$$\frac{dM_{2b}}{dt} = \underbrace{M_{0b}\left(\frac{k_{m0ab}a_b^2}{x_{m0ab}^2 + a_b^2}\right)}_{\substack{\text{Differentiation}\\ \text{to M2}}} - \underbrace{M_{2b}\frac{k_{ee}E_e^4}{x_{ee}^4 + E_e^4}}_{\text{Leak into lung}} - \underbrace{k_{m2}M_{2b}}_{\text{Migration}} - \underbrace{\mu_{M2_b}M_{2b}}_{\text{Decay}} \quad (1)$$

$$\frac{dM_2}{dt} = \underbrace{M_0\left(\frac{k_{m0a}a^2}{x_{m0a}^2 + a^2}\right)}_{\substack{\text{Differentiation}\\ \text{to M2}}} + \underbrace{M_{2b}\frac{k_{ee}E_e^4}{x_{ee}^4 + E_e^4}}_{\text{Leak into lung}} + \underbrace{k_{m2}M_{2b}}_{\text{Migration}} - \underbrace{\mu_{M_2}M_2}_{\text{Decay}}$$

$$+ \underbrace{k_{man}(k_{anm1}ANM_1)}_{\substack{\text{M1 switch to M2}\\ \text{by phagocytosis}}} \underbrace{\left(\frac{1}{1 + \left(\frac{a}{a_\infty}\right)^2}\right)}_{\substack{\text{Inhibition}\\ \text{by AIMs}}} \quad (2)$$
