# Supplementary material for: Age-dependent ventilator-induced lung injury: Mathematical modeling, experimental data, and statistical analysis
Source: PLoS Comput Biol. 2024 Feb 22;20(2):e1011113. doi: 10.1371/journal.pcbi.1011113 (PMC10914268; doi:10.1371/journal.pcbi.1011113)
Supplement: S4 Eq — (PDF) [file pcbi.1011113.s005.pdf]

S4 Eq. Neutrophil equations

$$\frac{dN_{0b}}{dt} = -N_{0b}\underbrace{\left(\frac{k_{n0pb}p_b^2}{x_{n0pb}^2+p_b^2}\right)}_{\text{Activation by PIMs}}\underbrace{\left(\frac{1}{1+\left(\frac{a_b}{a_{b\infty}}\right)^2}\right)}_{\text{Inhibition by AIMs}} + \underbrace{s_N}_{\text{Source}} \underbrace{-N_{0b}\frac{k_{ee}E_e^4}{x_{ee}^4+E_e^4}}_{\text{Leak into lung}} - \underbrace{\mu_{N_{0b}}N_{0b}}_{\text{Decay}} \tag{1}$$

$$\frac{dN_0}{dt} = \underbrace{N_{0b}\frac{k_{ee}E_e^4}{x_{ee}^4+E_e^4}}_{\text{Leak into lung}} - N_0\underbrace{\left(\frac{k_{n0p}p^2}{x_{n0p}^2+p^2}\right)}_{\text{Activation by PIMs}}\underbrace{\left(\frac{1}{1+\left(\frac{a}{a_{\infty}}\right)^2}\right)}_{\text{Inhibition by AIMs}} - \underbrace{\mu_{N_0}N_0}_{\text{Decay}} \tag{2}$$

$$\frac{dN_b}{dt} = N_{0b}\underbrace{\left(\frac{k_{n0pb}p_b^2}{x_{n0pb}^2+p_b^2}\right)}_{\text{Activation by PIMs}}\underbrace{\left(\frac{1}{1+\left(\frac{a_b}{a_{b\infty}}\right)^2}\right)}_{\text{Inhibition by AIMs}} - \underbrace{k_nN_b}_{\text{Migration}} \underbrace{-N_b\frac{k_{ee}E_e^4}{x_{ee}^4+E_e^4}}_{\text{Leak into lung}} - \underbrace{\mu_{N_b}N_b}_{\text{Decay}} \tag{3}$$

$$\begin{aligned}\frac{dN}{dt} = {} & \underbrace{k_nN_b}_{\text{Migration}} - \underbrace{k_{an}N}_{\text{Transition to apoptotic}} + N_b\underbrace{\frac{k_{ee}E_e^4}{x_{ee}^4+E_e^4}}_{\text{Leak into lung}} \\ & + N_0\underbrace{\left(\frac{k_{n0p}p^2}{x_{n0p}^2+p^2}\right)}_{\text{Activation by PIMs}}\underbrace{\left(\frac{1}{1+\left(\frac{a}{a_{\infty}}\right)^2}\right)}_{\text{Inhibition by AIMs}} - \underbrace{\mu_nN}_{\text{Decay}}\end{aligned} \tag{4}$$

$$\frac{dAN}{dt} = \underbrace{k_{an}N}_{\text{Transition to apoptotic}} - \underbrace{k_{anm1}ANM_1}_{\text{Phagocytosis by M1}}\underbrace{\left(\frac{1}{1+\left(\frac{a}{a_{\infty}}\right)^2}\right)}_{\text{Inhibition by AIMs}} - \underbrace{k_{anm2}ANM_2}_{\text{Phagocytosis by M2}} - \underbrace{\mu_{AN}AN}_{\text{Decay}} \tag{5}$$
