# Supplementary material for: Age-dependent ventilator-induced lung injury: Mathematical modeling, experimental data, and statistical analysis
Source: PLoS Comput Biol. 2024 Feb 22;20(2):e1011113. doi: 10.1371/journal.pcbi.1011113 (PMC10914268; doi:10.1371/journal.pcbi.1011113)
Supplement: S5 Eq — (PDF) [file pcbi.1011113.s006.pdf]

S5 Eq. Pro- and anti-inflammatory mediators equations

$$\frac{dp_b}{dt} = \underbrace{d_p(p - p_b)}_{\text{Diffusion}} + \underbrace{k_{pm1}M_{1b}}_{\substack{\text{Production}\\ \text{via M1}}} \underbrace{\left(\frac{1}{1 + \left(\frac{a_b}{a_{b\infty}}\right)^2}\right)}_{\substack{\text{Inhibition}\\ \text{by AIMs}}} + \underbrace{k_{pn}N_b}_{\substack{\text{Production via}\\ \text{neutrophils}}} + \underbrace{s_p}_{\substack{\text{Background}\\ \text{production}}} \underbrace{-p_b\frac{k_{ee}E_e^4}{x_{eem}^4 + E_e^4}}_{\text{Leak into lung}} - \underbrace{\mu_{p_b}p_b}_{\text{Decay}} \tag{1}$$

$$\frac{dp}{dt} = -\underbrace{d_p(p - p_b)}_{\text{Diffusion}} + \underbrace{k_{pm1}M_1}_{\substack{\text{Production}\\ \text{via M1}}} \underbrace{\left(\frac{1}{1 + \left(\frac{a}{a_{\infty}}\right)^2}\right)}_{\substack{\text{Inhibition}\\ \text{by AIMs}}} + \underbrace{k_{pn}N}_{\substack{\text{Production via}\\ \text{neutrophils}}} + \underbrace{k_{pe}E_d}_{\substack{\text{Production via}\\ \text{ep. damage}}} \underbrace{+p_b\frac{k_{ee}E_e^4}{x_{eem}^4 + E_e^4}}_{\text{Leak into lung}} - \underbrace{\mu_p p}_{\text{Decay}} \tag{2}$$

$$\frac{da_b}{dt} = \underbrace{d_a(a - a_b)}_{\text{Diffusion}} + \underbrace{k_{am1}M_{1b}}_{\substack{\text{Production}\\ \text{via M1}}} + \underbrace{k_{am2}M_{2b}}_{\substack{\text{Production}\\ \text{via M2}}} + \underbrace{s_a}_{\substack{\text{Background}\\ \text{production}}} \underbrace{-a_b\frac{k_{ee}E_e^4}{x_{eem}^4 + E_e^4}}_{\text{Leak into lung}} - \underbrace{\mu_{a_b}a_b}_{\text{Decay}} \tag{3}$$

$$\frac{da}{dt} = \underbrace{-d_a(a - a_b)}_{\text{Diffusion}} + \underbrace{k_{am1}M_1}_{\substack{\text{Production}\\ \text{via M1}}} + \underbrace{k_{am2}M_2}_{\substack{\text{Production}\\ \text{via M2}}} \underbrace{+a_b\frac{k_{ee}E_e^4}{x_{eem}^4 + E_e^4}}_{\text{Leak into lung}} - \underbrace{\mu_a a}_{\text{Decay}} \tag{4}$$
