# Supplementary material for: Age-dependent ventilator-induced lung injury: Mathematical modeling, experimental data, and statistical analysis
Source: PLoS Comput Biol. 2024 Feb 22;20(2):e1011113. doi: 10.1371/journal.pcbi.1011113 (PMC10914268; doi:10.1371/journal.pcbi.1011113)
Supplement: S6 Eq — (PDF) [file pcbi.1011113.s007.pdf]

S6 Eq. Repair and epithelial equations

$$\frac{dR}{dt} = \underbrace{k_{rm2}M_2}_{\substack{\text{Upregulation}\\\text{by M2}}} - \underbrace{\mu_R R}_{\text{Decay}} \quad (1)$$

$$\frac{dE_h}{dt} = \underbrace{(b_p + k_{ep}p)(E_h + E_d)E_e}_{\substack{\text{Proliferation of healthy cells,}\\\text{upregulated by PIMs}}} + E_d\left(\underbrace{b_r}_{\substack{\text{Baseline}\\\text{repair}}} + \underbrace{\frac{k_{er}R}{x_{er} + R}}_{\substack{\text{Upregulation}\\\text{via repair}\\\text{mediators}}}\right)$$
$$- \underbrace{Eh\left(\frac{k_{mne}(M_1 + N)^2}{x_{mne}^2 + (M_1 + N)^2}\right)}_{\substack{\text{Damage via}\\\text{M1 \& neutrophils}}} - \underbrace{s_d E_h}_{\substack{\text{Damage from}\\\text{ventilator}}} \quad (2)$$

$$\frac{dE_d}{dt} = - E_d\left(\underbrace{b_r}_{\substack{\text{Baseline}\\\text{repair}}} + \underbrace{\frac{k_{er}R}{x_{er} + R}}_{\substack{\text{Upregulation}\\\text{via repair}\\\text{mediators}}}\right) + \underbrace{E_h\left(\frac{k_{mne}(M_1 + N)^2}{x_{mne}^2 + (M_1 + N)^2}\right)}_{\substack{\text{Damage via}\\\text{M1 \& neutrophils}}}$$
$$- \underbrace{k_{em1}M_1E_d}_{\substack{\text{Phagocytosis}\\\text{of damaged}\\\text{cells by M1}}}\underbrace{\left(\frac{1}{1 + \left(\frac{a}{a_\infty}\right)^2}\right)}_{\substack{\text{Inhibition}\\\text{by AIMs}}} - \underbrace{k_{en}NE_d}_{\substack{\text{Phagocytosis}\\\text{of damaged}\\\text{cells by N}}} + \underbrace{s_d E_h}_{\substack{\text{Damage from}\\\text{ventilator}}} - \underbrace{b_d E_d}_{\text{Death}} \quad (3)$$

$$\frac{dE_e}{dt} = - \underbrace{(b_p + k_{ep}p)(E_h + E_d)E_e}_{\substack{\text{Proliferation of healthy cells,}\\\text{upregulated by PIMs}}}$$
$$+ \underbrace{k_{em1}M_1E_d}_{\substack{\text{Phagocytosis}\\\text{of damaged}\\\text{cells by M1}}}\underbrace{\left(\frac{1}{1 + \left(\frac{a}{a_\infty}\right)^2}\right)}_{\substack{\text{Inhibition}\\\text{by AIMs}}} + \underbrace{k_{en}NE_d}_{\substack{\text{Phagocytosis}\\\text{of damaged}\\\text{cells by N}}} + \underbrace{b_d E_d}_{\text{Death}} \quad (4)$$
